# Genome-Wide Identification and Analysis of the AP2 Transcription Factor Gene Family in Wheat (*Triticum aestivum* L.)

**DOI:** 10.3389/fpls.2019.01286

**Published:** 2019-10-11

**Authors:** Yue Zhao, Renyi Ma, Dongliang Xu, Huihui Bi, Zongliang Xia, Huiru Peng

**Affiliations:** ^1^College of Life Science, Henan Agricultural University, Zhengzhou, China; ^2^College of Agronomy/Collaborative Innovation Center of Henan Grain Crops/National Key Laboratory of Wheat and Maize Crop Science, Henan Agricultural University, Zhengzhou, China; ^3^State Key Laboratory for Agrobiotechnology, Key Laboratory of Crop Heterosis and Utilization (MOE), Beijing Key Laboratory of Crop Genetic Improvement, China Agricultural University, Beijing, China

**Keywords:** AP2, *Arabidopsis*, expression pattern, organ size, phylogenetic tree, transgenic, wheat

## Abstract

The AP2 transcription factors play important roles in regulating plant growth and development. However, limited data are available on the contributions of AP2 transcription factors in wheat (*Triticum aestivum* L.). In the present study, a total of 62 *AP2* genes were identified in wheat from a genome-wide search against the latest wheat genome data. Phylogenetic and sequence alignment analyses divided the wheat *AP2* genes into 3 clusters, euAP2, euANT, and basalANT. Chromosomal distribution, gene structure and duplication, and motif composition were subsequently investigated. The 62 *TaAP2* genes were unevenly distributed on 21 chromosomes. Twenty-four homologous gene sets among A, B, and D sub-genomes were detected, which contributed to the expansion of the wheat *AP2* gene family. The expression levels of *TaAP2* genes were examined using the WheatExp database; most detected genes exhibited tissue-specific expression patterns. The transcript levels of 9 randomly selected *TaAP2* genes were validated through qPCR analyses. Overexpression of *TaAP2-10-5D*, the most likely homolog of *Arabidopsis* *ANT* gene, increased organ sizes in *Arabidopsis*. Our results extend our knowledge of the *AP2* gene family in wheat, and contribute to further functional characterization of *AP2s* during wheat development with the ultimate goal of improving crop production.

## Introduction

The APETALA2/Ethylene-Responsive Factor (AP2/ERF) superfamily is one of the largest groups of transcription factors (TFs) in plants. The AP2/ERF superfamily is defined by the conserved AP2 DNA binding domains of 57–66 amino acids (Okamuro et al., 1997). In general, the AP2/ERF superfamily is divided into three separate families, which are AP2, ERF, and RAV families, based on the number of AP2 domains and sequence similarities. The AP2 family proteins contain two repeated AP2 domains; the ERF family proteins contain a single AP2 domain; the RAV (related to ABI3/VP1) family proteins contain two different DNA binding domains, AP2 and B3 (Sakuma et al., 2002; Nakano et al., 2006). The AP2 family was subdivided into the euAP2, eu-AINTEGUMENTA (euANT) and basalANT groups according to the amino acid sequence of the double AP2 domain and the nuclear localization sequence (Shigyo et al., 2006; Wang et al., 2016a; Dipp-Alvarez and Cruz-Ramirez, 2019).

The AP2 family genes play key roles in the reproductive and vegetative organs development (Gil-Humanes et al., 2009; Wang et al., 2016a; Scheres and Krizek, 2018). In Arabidopsis, 18 AP2 family genes have been identified (Sakuma et al., 2002). Arabidopsis AP2 is the most well-studied gene in the AP2 family. AtAP2 gene plays a central role in the specification of floral organ identities (Bowman et al., 1989; Kunst et al., 1989), the regulation of flowering time and floral meristem (Coen and Meyerowitz, 1991), the control of floral homeotic gene expression (Drews et al., 1991; Mandel et al., 1992), and the modulation of seed development (Jofuku et al., 2005) in Arabidopsis. The *TARGETS OF EAT (TOE)* genes, homologs of *AtAP2,* were shown to affect flowering time in Arabidopsis by repressing the expression of flowering regulatory genes (Mathieu et al., 2009; Yant et al., 2010). The *TOE* genes include *TOE1, TOE2, TOE3, SCHLAFMÜTZE (SMZ), and SCHNARCHZAPFEN (SNZ)*; these genes are negatively regulated by *microRNA172* (*miR172*) at the posttranscriptional level (Aukerman and Sakai, 2003; Chen, 2004; Zhang et al., 2015b). In addition, the *ANT, ANT-LIKE5 (AIL5)*, *AIL6*, and *AIL7* genes, members of the euANT group of the AP2 family, regulate ovule development and floral organ growth (Elliott et al., 1996; Klucher et al., 1996; Nole-Wilson et al., 2005; Krizek, 2009; Krizek, 2015). Another members of the euANT group, *AtBBM* and *PLT* genes, were reported to likely function during embryogenesis (Boutilier et al., 2002), and function in roots (Aida et al., 2004), respectively. In rice (*Oryza sativa* L.), 23 *AP2* members have been identified (Rashid et al., 2012), and some of them have been functionally characterized, including *SMALL ORGAN SIZE1* (*SMOS1*) (Aya et al., 2014), *SUPERNUMERARY BRACT (SNB)* and *INDETERMINATE SPIKELET1* (*OsIDS1*) (Lee and An, 2012; Lee et al., 2014; Jiang et al., 2019). *SNB* and *OsIDS1* together play important roles in inflorescence architecture and the establishment of floral meristems (Lee and An, 2012). Recently, it was found that SNB controls rice seed shattering and seed size (Jiang et al., 2019). Maize IDS1 is expressed in roots, leaves, inflorescence primordia, spikelet pair primordia, and embryos (Chuck et al., 2008). In the *ids1* mutant, the spikelet meristem becomes indeterminate and produces additional florets (Chuck et al., 2008). It was also reported that maize AP2-like gene *glossy15* regulates leaf epidermal cell identity (Moose and Sisco, 1996) and *kernel row number 1 (krn1)* enhanced kernel row numbers (Wang et al., 2019a).

Wheat (*Triticum aestivum* L., 2n = 6x = 42, BBAADD) is one of the most important crops in the world. Extensive research has been conducted on the AP2/ERF superfamily transcription factors in wheat, but attention has mainly focused on the ERF family because of their significant roles in abiotic and biotic stresses (Xu et al., 2011; Kulkarni et al., 2017). Up to now, only three AP2 family genes (*Q*, *TaAP2*, and *TaPARG*) have been characterized in wheat. Gene *Q* has pleiotropic influences on many traits, such as grain and spike morphology, plant height, and spike emergence time (Simons et al., 2006; Greenwood et al., 2017; Liu et al., 2018; Xie et al., 2018; Xu et al., 2018). *TaAP2*, the gene underlying lodicule development, is required for engineering of cleistogamous wheat (Ning et al., 2013). Li et al. (2016) reported that *TaPARGs* play a key role in growth and development, including regulation of plant architecture-related traits and yield-related traits. To further explore the roles of AP2 genes in wheat, we performed a genome-wide search of AP2 family genes using the currently released wheat genome data. Phylogenetic, chromosomal location, gene structure, and expression pattern analyses of the identified wheat AP2 genes were undertaken. One of the family members designated *TaAP2-10* was demonstrated to play an important role in regulating organ size. These results contribute to enrich our knowledge of AP2 gene family in wheat, and lay a basis for future functional analysis of the wheat AP2 family genes.

## Materials and Methods

### Database Search and Physical Locations of *AP2s* in Wheat

The wheat genome sequencing has been completed in 2018 (Appels et al., 2018), and filtered protein and coding sequences have also become available^1^. First, all the wheat AP2 domain-containing protein sequences were downloaded from the Ensembl Plants database^2^ after a hidden Markov model (HMM) search using the HMM profiles of the AP2 domain (Pfam ID: PF00847) as queries. Then, the sequences were checked by CDD^3^ (Marchler-Bauer et al., 2017) to confirm the presence of the AP2 domain. Furthermore, all candidate sequences were analyzed by means of SMART^4^ (Letunic et al., 2015) and PFAM databases^5^ to eliminate the sequences not containing two AP2 domains. As a final quality check, the existence of all candidate sequences were examined by BLASTN similarity search against the wheat ESTs deposited in NCBI database^6^. The isoelectric points (*p*I) and protein molecular weights (MW) of the putative TaAP2s were calculated using the ExPASy online tool^7^. The physical locations of all designated AP2 genes were obtained from the URGI IWGSC BLAST^8^. The chromosomal location image was generated by the MapInspect software^9^. *Arabidopsis* AP2 sequences were downloaded from The *Arabidopsis* Information Resources (TAIR^10^) and rice AP2 sequences were from Rice Genome Annotation Project (RGAP^11^) (Kawahara et al., 2013).

### Gene Structure, Multiple Sequence Alignment, and Construction of Phylogenetic Tree

The exon/intron structures were constructed by GSDS^12^ (Hu et al., 2015) using the coding sequences (CDS) and corresponding genomic sequences retrieved from the Ensembl Plants database^2^. Multi-sequence alignments were carried out using the ClustalW version 2.0 with default settings (Larkin et al., 2007). Phylogenetic and molecular evolutionary analysis was conducted by MEGA version 6 using the Neighbor-Joining and Maximum-Likelihood methods (Tamura et al., 2013). The reliability of phylogenetic trees was tested using bootstrapping with 1,000 replicates.

### Analysis of Gene Duplication

To identify duplicated gene pairs, we defined gene duplication according to the following criteria (Wang et al., 2016b): (1) the alignable nucleotide sequence covered was >80% of the longer aligned gene, and (2) the aligned region had an identity >80%.

### Motif Analysis

MEME^13^ (Multiple Em for Motif Elicitation, version 4.11.4) was used to identify conserved motifs within complete AP2 protein sequences of wheat using the following parameters: optimum motif width set to ≥6 and ≤50; maximum number of motifs: 16.

### Analysis of Gene Expression Profiles

Gene expression data of *TaAP2* genes in different wheat tissues (spike, root, leaf, grain, and stem) of the hexaploid bread wheat (var. Chinese Spring) were obtained from the WheatExp database^14^ (Pearce et al., 2015). The tissues include leaves at seedling and reproductive stages, roots at seedling and reproductive stages, young spikes, spikes at flowering, stems at anthesis stage, and 14 days after anthesis (DAA) grains. The expression patterns were presented as a heat map, which reflected the FPKM (Fragments Per Kilobase of transcript per Million mapped reads), with red indicating high expression levels, yellow indicating medium expression levels, and blue indicating almost no expression. The heat map was plotted using the Heatmap Illustrator HemI v.1.0 (Deng et al., 2014).

### Plant Materials and Growth Conditions

Seeds of var. Chinese Spring were grown under controlled glass-house conditions. Roots, stems, leaves, developing spikes (length: 10–20 mm), and spikes at flowering were collected from three wheat plants, and all of the samples were immediately frozen in liquid nitrogen and then stored at −80°C for RNA extraction.

The *Arabidopsis* Col-0 was used to generate *TaAP2-10-5D* transgenic plants. The *Arabidopsis* plants were grown under controlled conditions in a greenhouse at 22°C with 60% relative humidity and the photoperiod of 16 h light and 8 h dark at 110 µmol m^−2^s^−1^ or on half-strength Murashige and Skoog (MS) medium. After grown for 4 weeks on soil, images of each plant were taken from the above, and used for rosette diameter measurements using ImageJ software^15^. Plant height and fresh weight were recorded at the mature period. The silique length was measured from the three longest siliques of each plant. A total of 10–20 plants per genotype were analyzed in each experiment.

### RNA Extraction and qPCR Analysis

Total RNA was extracted using TRIzol reagent (Invitrogen, USA). The isolated RNA (2 μg) was treated with DNase I (TaKaRa, Japan), and cDNA synthesis was conducted using the 5X All-In-One RT MasterMix (abm, Canada) according to the manufacturer’s protocol. Real-time quantitative PCR (qPCR) was performed on StepOne^™^ and StepOnePlus^™^ Real-Time PCR Systems (Life Technologies, USA) using the SYBR^®^ Green reaction kit (TaKaRa, Japan). The wheat β-actin and *AtActin2* were used as internal reference genes in wheat and *Arabidopsis*, respectively. The conditions for qPCR have been described previously (Zhao et al., 2018). Three biological replicates and three technical replicates were applied for all qPCR analyses in this study. The relative gene expression levels were calculated using the 2^−ΔCt^ method (Livak and Schmittgen, 2001). The primers used for the qPCR analysis were listed in **Table S1**.

### Generation of Transgenic Plants

The *TaAP2-10-5D* CDS was amplified through PCR using primers TaAP2-10-tDF/R, and subsequently subcloned into the pSuper1300 plant expression vector harbouring the *mannopine synthase* (*mas*) promoter. The transformation of the obtained recombinant vector into wild type (Col-0) *Arabidopsis* was performed *via Agrobacterium tumefaciens* (strain GV3101) mediated floral-dip method (Clough and Bent, 1998). The transgenic plants were screened by 0.1% Hygromycin B solution and then confirmed by qPCR.

### Microscopy Counts of Leaf Epidermal Cells

The fifth leaf was collected from greenhouse-grown 4-week-old wheat plants (n = 5) for counts of leaf epidermal cells, as described previously (Zhao et al., 2017). Briefly, chlorophyll was removed by washing the leaf 2–3 times until the washing solution (70% ethanol) remained clear. The leaf epidermis was peeled off and mounted on a glass slide under a cover glass. Images were taken using a Nikon Ti-U microscope. All whole cells captured in the view field were counted.

## Results

### Identification of AP2 Gene Family in Wheat

To identify wheat *AP2* genes, a HMM search was conducted using the HMM profiles of the AP2 domain (Pfam ID: PF00847) as queries against the latest genome data of wheat in the Ensembl Plants database. A total of 565 sequences were discovered as potentially encoding AP2 domain(s)-containing proteins in the wheat genome. Subsequently, all putative genes were examined to check the number of AP2 domains in the encoded proteins using the SMART program. We found that the protein sequences of 62 genes contain two AP2 domains, while the remaining 503 sequences contain a single or partial AP2 domain. Detailed information on the 62 *AP2* genes was listed in **Table S2**. The lengths of TaAP2 proteins ranged from 328 (TaAP2-18-4D) to 703 amino acids (TaAP2-3-3D). The isoelectric points (*p*I) ranged from 5.16 (TaAP2-19-6D) to 9.47 (TaAP2-15-6A).

### Classification and Phylogenetic Analyses of TaAP2s

**Figure 1:**
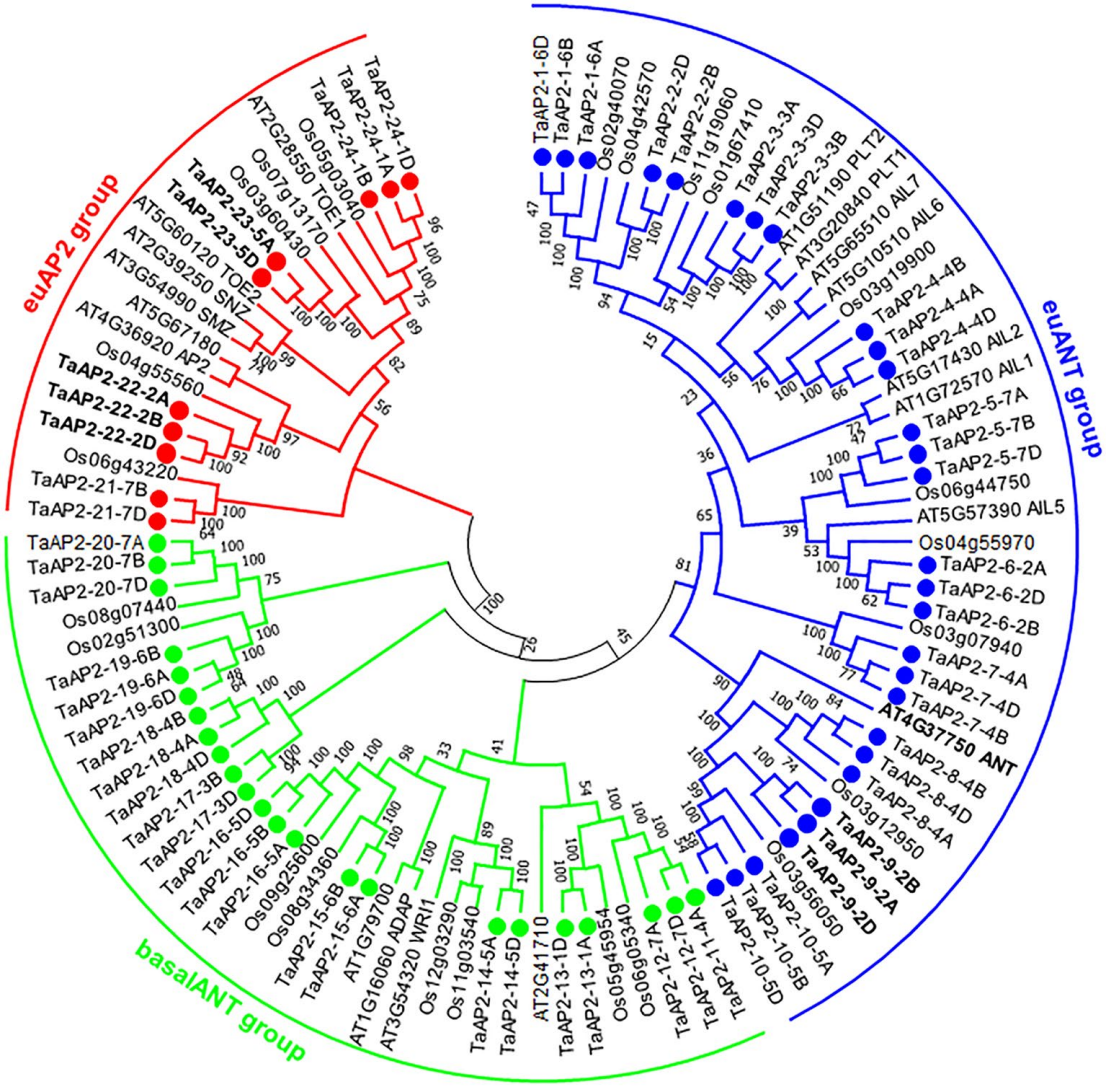
Phylogenetic tree of AP2 proteins from wheat, *Arabidopsis*, and rice. The unrooted neighbor-joining (NJ) tree was constructed based on the AP2 proteins from wheat (62), *Arabidopsis* (18), and rice (23) using MEGA6.0. The numbers at nodes are bootstrap values after 1000 repetitions. The wheat AP2 TF proteins are named according to their positions in the tree. Each TaAP2 protein is indicated by a dot. The wheat AP2 proteins were classified into three groups, group euAP2, euANT, and basalANT, and they are represented by red, blue, and green, respectively. The three characterized *TaAP2* genes and *Arabidopsis ANT* are highlighted in bold (*TaAP2-23*: Q; *TaAP2-22*: *TaAP2*; and *TaPARG*: *TaAP2-9*).

In order to precisely reveal the evolutionary relationships of the TaAP2 proteins, we performed phylogenetic analyses of 103 AP2 proteins, including 62 wheat, 18 *Arabidopsis* and 23 rice AP2s using both neighbor-joining method (**Figure 1**) and maximum-likelihood algorithm (**Figure S1**). The genes from different wheat sub-genomes but in adjacent branches of phylogenetic trees were regarded as different copies of each member of the *TaAP2* gene family. Thus, we obtained 24 *wheat AP2* members designated from *TaAP2-1* to *TaAP2-24*, and the homologous copies of each member were distinguished by subjoining the wheat sub-genome symbols A, B or D. According to the previous classification of AP2 genes in *Arabidopsis* and rice (Kim et al., 2006; Shigyo et al., 2006), wheat *TaAP2* genes were also categorized as three types: euAP2, euANT, and basalANT. The bootstrap values are reasonably high for the derived subclades and for the euAP2 group; however, these support values are lower at more basal branches. For instance, the support value for one branch of basal ANT group and the euANT group is 45% (**Figure 1**). Low support values were also observed in the ANT groups in the ML tree (**Figure S1**). Therefore, it is difficult to differentiate the basalANT group from the euANT group based on phylogenetics. Next, we conducted a sequence alignment of all the 24 members of wheat AP2 proteins. As expected, all the wheat proteins in the putative euANT group contain euANT1-4 motifs (**Figure S2**), which are exclusively present in the euANT group in plants (Kim et al., 2006). Finally, 10, 29, and 23 genes were assigned to euAP2, euANT, and basalANT groups, respectively.

### Motif Composition and Gene Structure Analysis of *TaAP2* Genes

**Figure 2:**
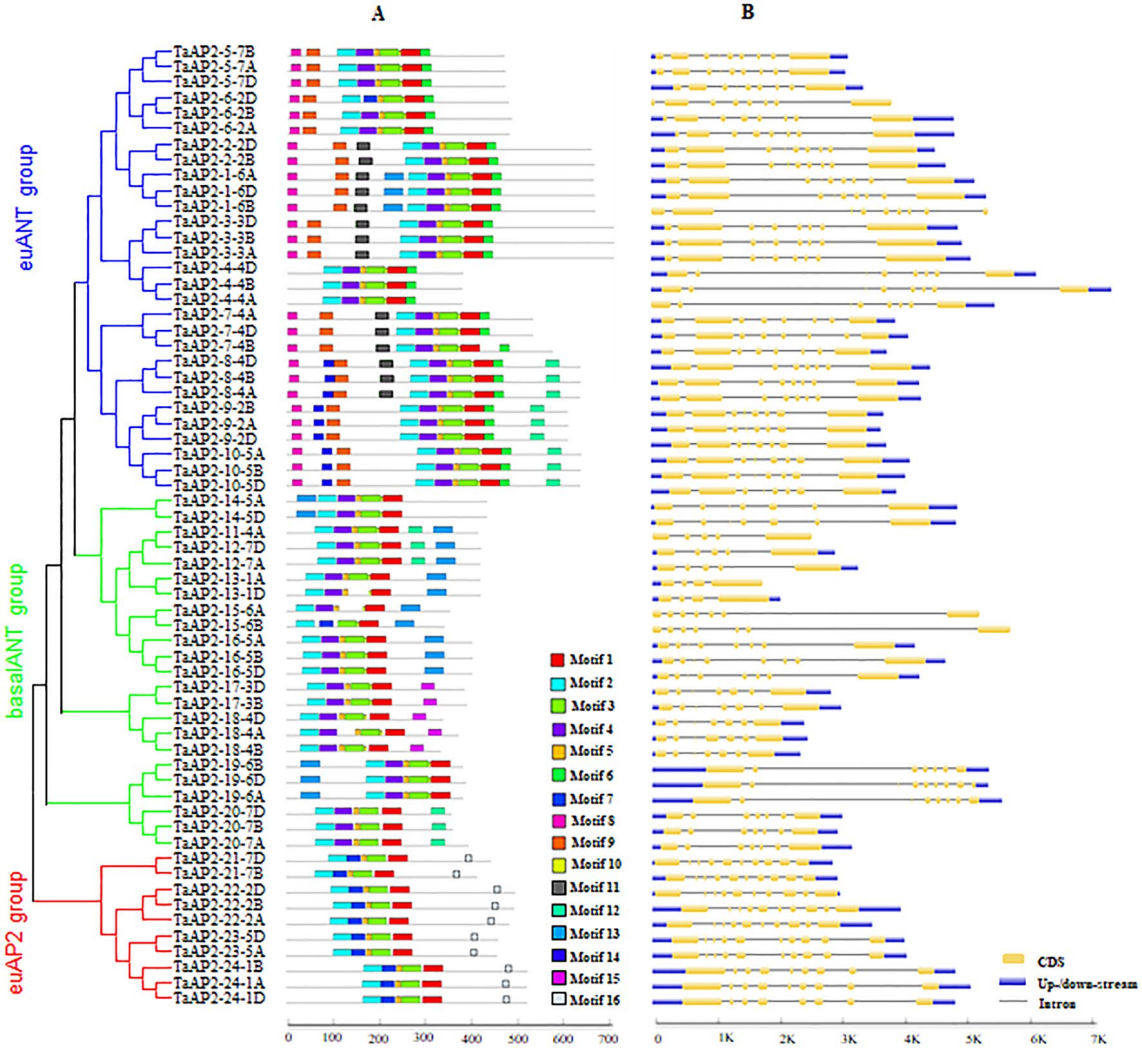
Motif composition and exon–intron structures of TaAP2s. **(A)** Conserved motifs organization of TaAP2 proteins. Color blocks represent the position of motifs on corresponding proteins. Block sizes indicate the lengths of motifs. Gray lines represent non-conserved sequences. The relative position of each motif can be determined using the scale below. **(B)** Exon–intron structures of *TaAP2* genes. Exons are represented by yellow boxes and introns were denoted by blank lines. The lengths of exons and introns could be estimated using the scale below.

MEME was used to analyze protein motifs in the 62 AP2 protein sequences in wheat (**Figure 2A**). In total, 16 conserved motifs were identified and designated motif 1–16. Motifs 1, 2, 3 and 5 were found in almost all the members of the AP2 family. Among them, Motifs 1 and 3 were parts of one AP2 domain, while motifs 2 and 5 were components of the other AP2 domain. The three groups of AP2 family proteins had their unique motif organizations. The euAP2 group had two motifs unique to its group, which were motifs 7 and 16. In groups euANT and basalANT, motif 7 was replaced by motif 4, both of which were located on one AP2 domain. Motif 16 contained the predicted amino acid sequences (AAASSGF[S/P]) of the miR172 binding site. The majority of proteins in the basalANT group had the motif 13 and the remaining members in this group had motif 15, and both of them were distinctive to the group. Motifs 6, 8 and 9 were exclusively contained in the proteins of the euANT group. The consensus sequences of these motifs are given in **Figure S3** and **Table S3**.

In order to obtain more insights about gene evolution, the exon–intron organization of *TaAP2* genes was investigated by aligning predicted coding sequences (CDS) against corresponding genomic sequences using the online service GSDS. As shown in **Figure 2B** and **Table S2**, the number of introns in the *TaAP2* family genes ranged from 3 to 9. Overall, highly similar gene structures were observed for the three groups of AP2 family genes, especially for euANT and euAP2 groups. The number of introns in the genes of the euANT and euAP2 groups were 7 or 8, and 8 or 9, respectively. Comparatively, the basalANT group exhibited diverse gene structures, with the number of introns ranging from 3 to 7. Noticeably, all homologous genes in the AP2 family had the same number of introns.

### Genomic Distribution and Gene Duplication of *TaAP2* Genes

**Figure 3:**
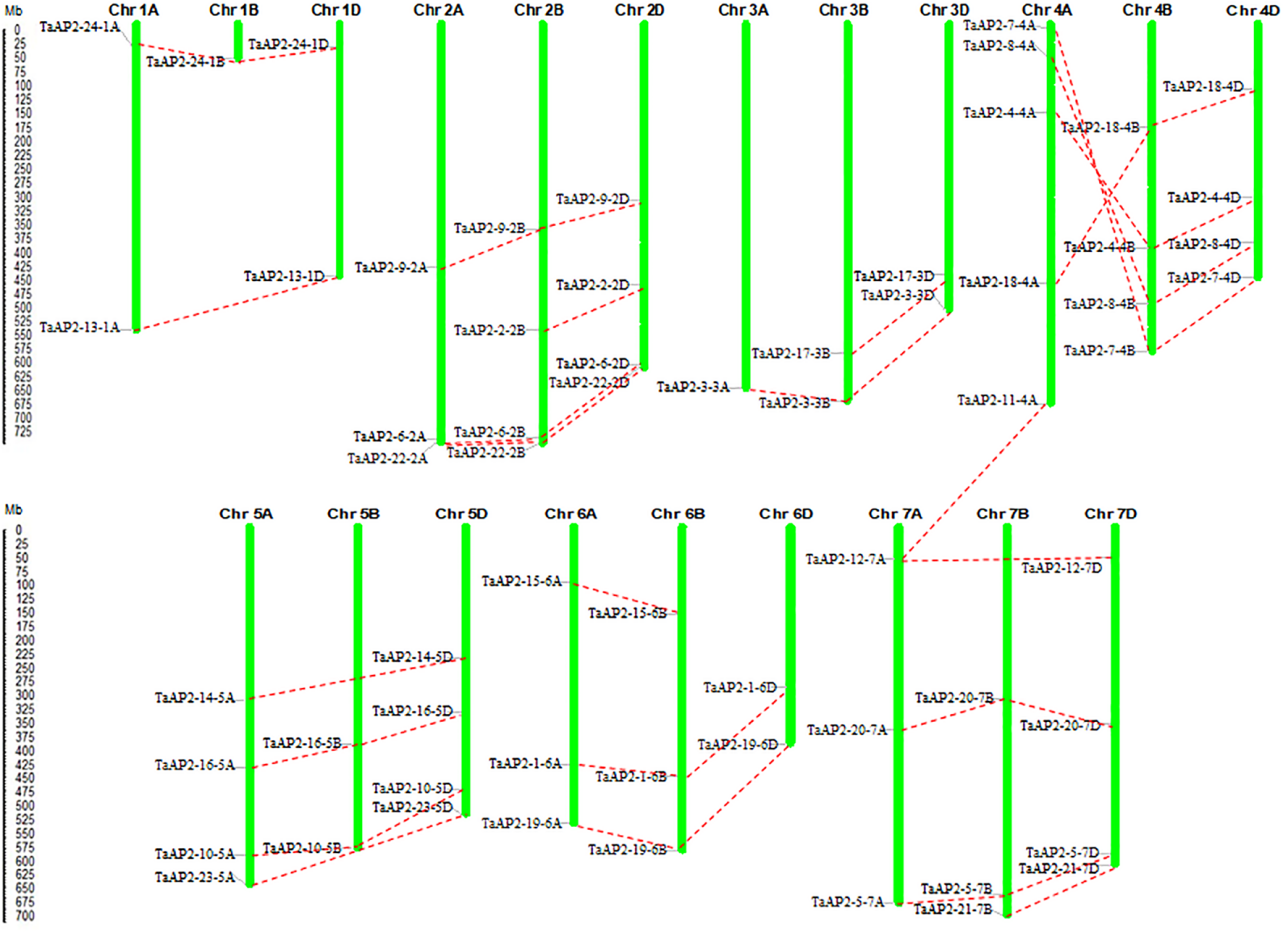
Locations and duplication of the *TaAP2* family genes on wheat chromosomes. The chromosomal position of each *TaAP2* was mapped according to the physical positions of wheat genomes (**Table S2**). The chromosome number is labeled at the top of each chromosome. The scale is in mega bases (Mb). Putative *TaAP2* homologous genes are depicted through dotted lines.

The *TaAP2* genes were distributed unevenly among the 21 chromosomes of the wheat genome (**Figure 3**). The number of genes on each chromosome ranged from 1 (chromosomes 1B and 3A) to 5 (chromosome 4A), with up to ten *TaAP2* genes located on each of chromosome groups 2, 4, 5, and 7. Among the 62 *TaAP2* genes, there were 21, 19, and 22 members distributed on wheat sub-genomes A, B, and D, respectively (**Figure 3**). In terms of gene duplication, there were 15 *TaAP2* members (*TaAP2-1, 3, 4, 5, 6, 7, 8*, *9*, *10*, *16*, *18*, *19*, *20*, *22, and 24*) containing three copies and eight *members* (*TaAP2-2, 12, 13, 14, 15, 17, 21, and 23*) containing two copies, and *TaAP2-11* had only one copy which was on chromosomes 4A (**Figure 3** and **Table S4**). Interestingly, TaAP2-11-4A had the highest sequence similarities to TaAP2-12-7A (95.3%) and TaAP2-12-7D (96.3%). Except for TaAP2-11-4A, no tandem or segmental duplication involving TaAP2s was discovered in the wheat genome. In addition, we found that *TaAP2* genes on chromosome 4A were reversed with their homologous genes on chromosomes 4B and 4D (**Figure 3**).

### Expression Profiles of *TaAP2* Genes in Various Wheat Tissues

**Figure 4:**
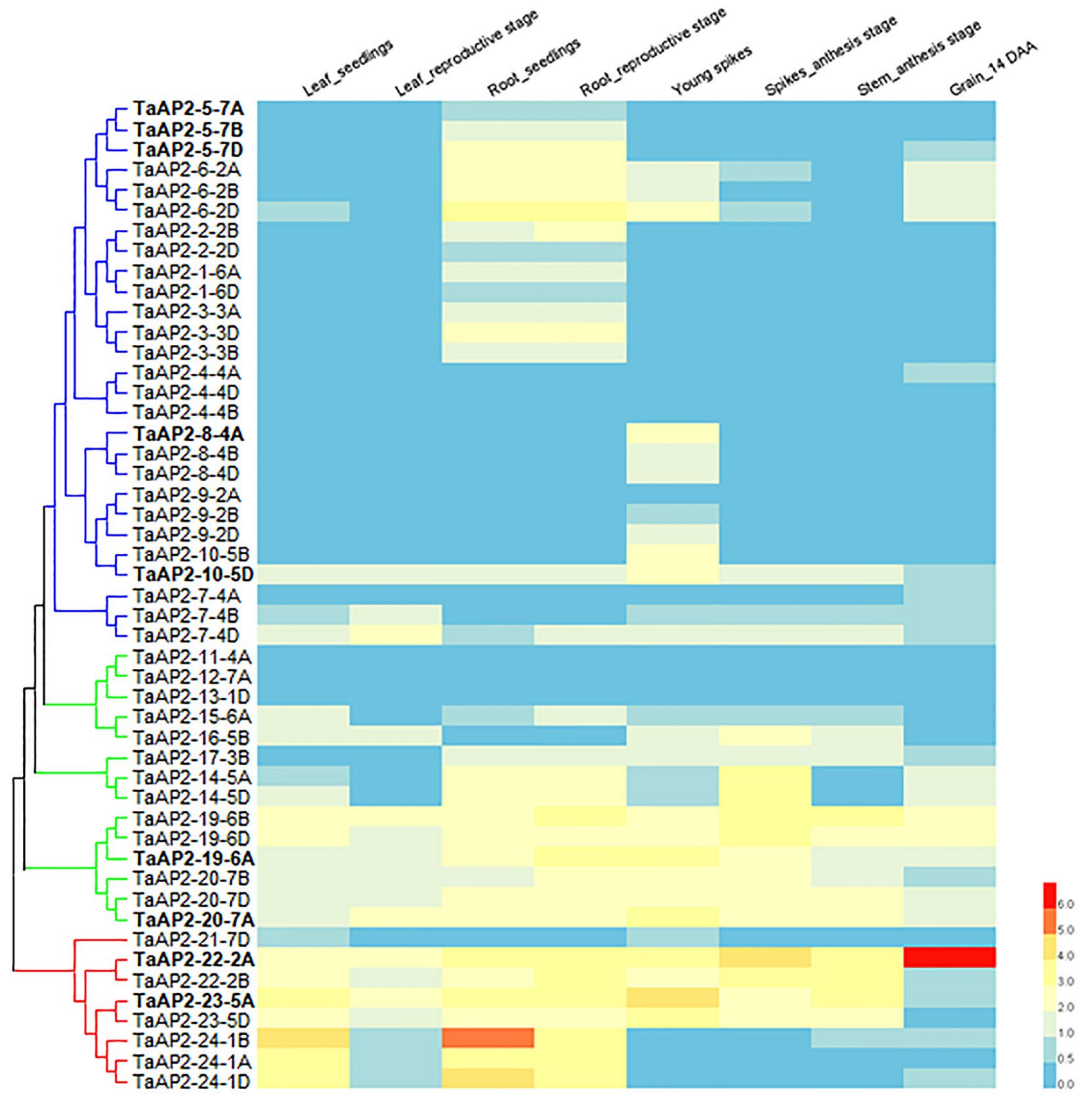
Expression patterns of 49 *TaAP2* genes in various tissues at different developmental stages in wheat. The tissues and developmental stages examined are indicated at the top of the heat map. Log_2_ transformed (FPKM + 1) expression values were used to create the heat map. The highest level of expression is represented by red (100%), while the low level is represented by blue (0%).

Gene expression pattern often has a strong correlation with gene functions. We investigated the expression levels of *TaAP2s* in various wheat tissues (leaf, root, spike, stem, and grain) across different developmental stages using the expression data in the WheatExp database. The expression data on 49 *TaAP2* genes were found in the database, while the information on the remaining 13 genes were missing (**Figure 4**). Of the 49 *TaAP2s*, 11 *TaAP2s* (*TaAP2-7-4D*, *TaAP2-10-5D*, *TaAP2-19-6A/B/D*, *TaAP2-20-7A/B/D*, *TaAP2-22-2A/B*, and *TaAP2-23-5A*) were expressed in all tissues at different developmental stages, whereas 6 *TaAP2s* (*TaAP2-4-4B/D*, *TaAP2-9-2A*, *TaAP2-11-4A*, *TaAP2-12-7A*, and *TaAP2-13-1D*) had almost no expression in any tested tissues (**Figure 4**). The remaining 32 *TaAP2s* were expressed in one or several specific tissue(s). The expression patterns of *TaAP2* genes had similarities and differences within a group. The majority of genes in the euAP2 and basalANT groups had relatively high levels of expression in all or most tissues examined, whereas most genes in the euANT group were mainly expressed in roots and spikes (**Figure 4**). In terms of the gene expression patterns among homologous genes, most homologous genes shared a similar expression pattern, including *TaAP2-5, -6, -2, -1, -3, -4, -8, -9, -14, -19, -20, -22, -23*, and -*24*. In contrast, different expression profiles were observed for *TaAP2-7* and *TaAP2-10*.

**Figure 5:**
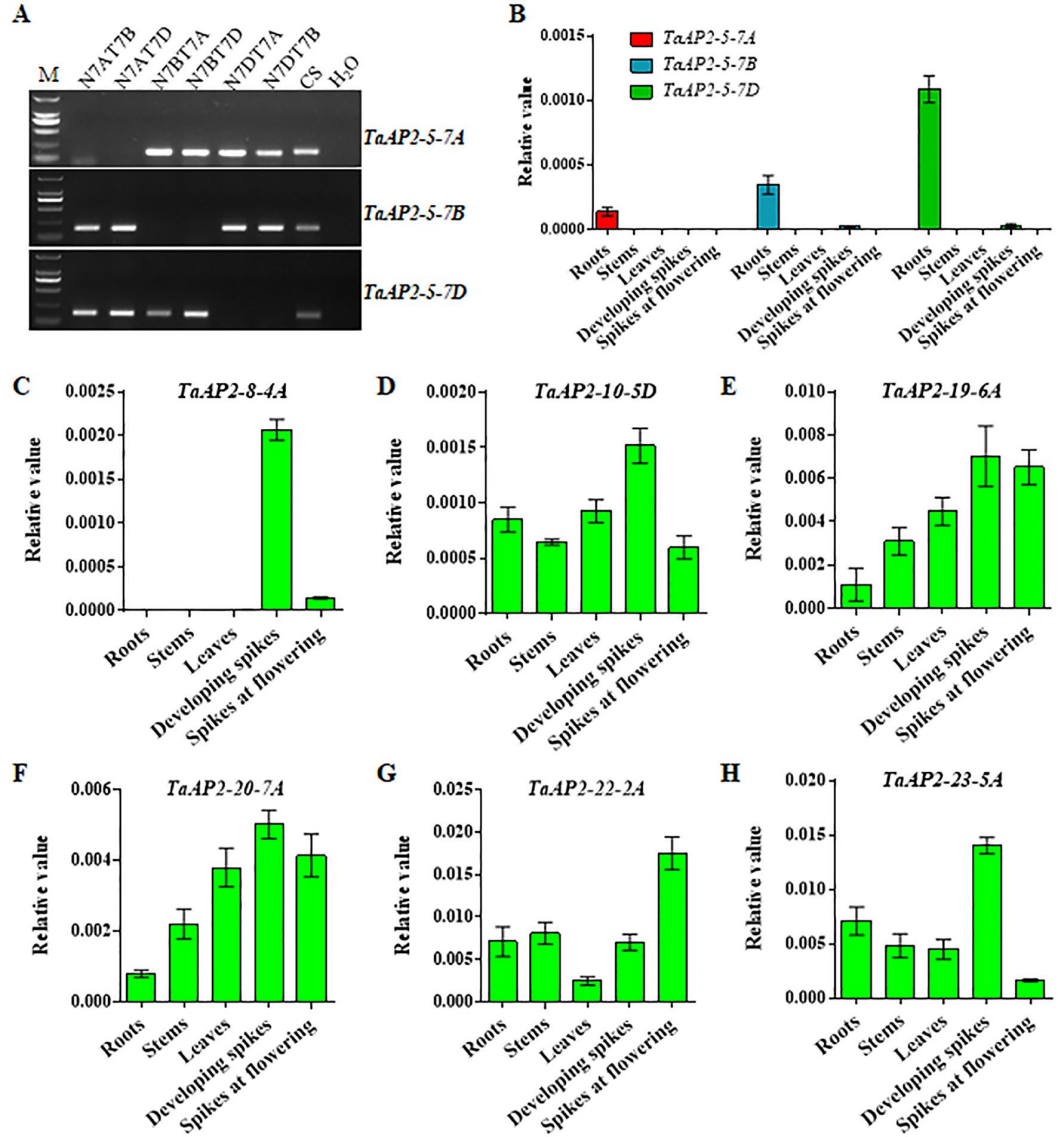
QPCR verification of the expression levels of several *TaAP2 *genes in different wheat tissues, including roots, stems, leaves, developing spikes, and spikes at flowering. **(A)** Verification of the sub-genome specificity of primer pairs for *TaAP2-5* genes in Chinese Spring nullisomic/tetrasomic (NT) lines. **(B)** Expression levels of *TaAP2-5A*, *TaAP2-5B*, and *TaAP2-5D* revealed by qPCR. **(C**–**H)** Expression levels of *TaAP2-8-4A* **(C)**, *TaAP2-10-5D* **(D)**, *TaAP2-19-6A* **(E)**, *TaAP2-20-7A* **(F)**, *TaAP2-22-2A* **(G)**, and *TaAP2-23-5A* **(H)** revealed by qPCR. Data were normalized by the wheat β-actin gene using the 2^−ΔCt^ method. Bars indicate standard deviations of three biological replicates.

To validate the gene expression profiles obtained from the WheatExp database, wheat roots, stems, leaves, and spikes were collected for RNA extraction and qPCR analysis. According to *TaAP2-5* gene sequences, we designed sub-genome-specific primer sets for *TaAP2-5A*, *TaAP2-5B* and *TaAP2-5D*. The specificity of each primer set was validated in Chinese Spring nullisomic/tetrasomic (NT) lines (**Figure 5A**). As shown in **Figure 5B**, *TaAP2-5s* was dominantly expressed in roots, and the expression level of *TaAP2-5D* was higher than those of *TaAP2-5B* and *TaAP2-5A*. The expression patterns of *TaAP2-5s* shown here resemble those in **Figure 4**. The consistent results between qPCR and public data were also observed for *TaAP2-8-4A*, *TaAP2-10-5D*, *TaAP2-19-6A*, *TaAP2-20-7A*, *TaAP2-22-2A*, and *TaAP2-23-5A* (**Figures 5C–H** and **Figure 4**).

### Overexpression of *TaAP2-10-5D* Led to Enlarged Plant Sizes in *Arabidopsis*

**Figure 6:**
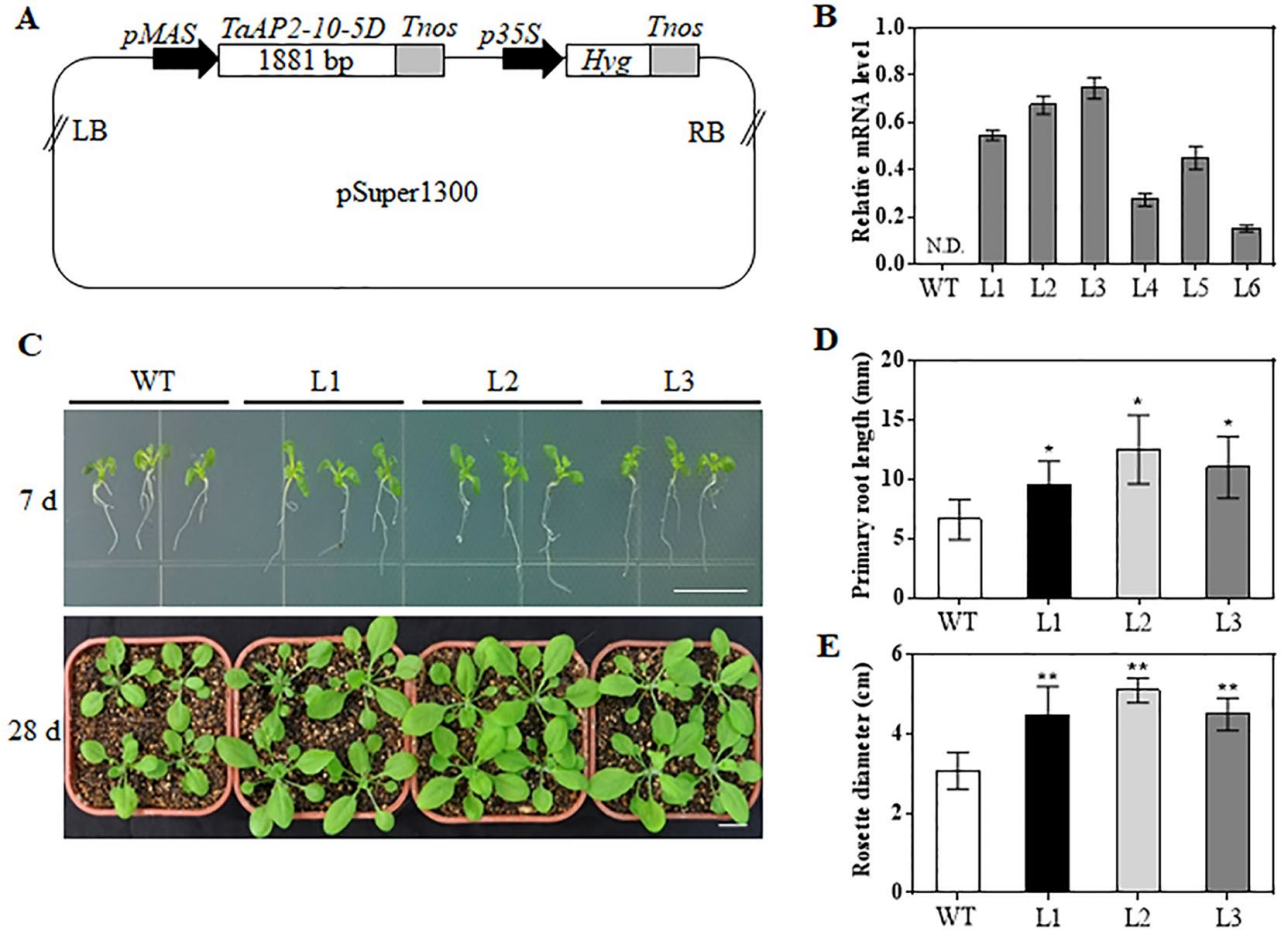
Morphological differences between WT and *TaAP2-10-5D* transgenic plants at seedling stages. **(A)** Schematic diagram of the construct containing the *TaAP2-10-5D* expression cassette. LB, left border; *pMAS*, *mannopine synthase* promoter; *Tnos*, *nopaline synthase gene* (*NOS*) terminator; *p35S*, *cauliflower mosaic virus 35S* promoter; *Hyg*, *Hygromycin-resistance gene*; RB, right border. **(B)** Transcript levels of *TaAP2-10-5D* in T_3_ transgenic *Arabidopsis* lines revealed by qPCR analysis. Bars indicate standard deviations of three biological replicates. N.D., Not Detected. **(C)** Phenotype of WT and *TaAP2-10-5D* transgenic seedlings grown for 7 and 28 days. **(D)** Root lengths of the WT and *TaAP2-10-5D* transgenic plants at the 7 day after sowing. **(E)** Rosette diameters of 28-day-old WT and *TaAP2-10-5D* transgenic plants. Bars = 10 mm. Mean values were calculated from 10–20 biological replicates, with error bars representing standard deviations. Statistically significant differences are indicated: *, *P* < 0.05; **, *P* < 0.01 (Student’s *t*-test).

TaAP2-10-5D was phylogenetically close to *Arabidopsis* ANT (AT4G37750) (**Figure 1**). Moreover, *TaAP2-10-5D* was ubiquitously expressed in different tissues, which was the same as *Arabidopsis* *ANT*. Thus, we hypothesized that *TaAP2-10-5D* might play an important role in growth and development of wheat. To uncover the potential function of *TaAP2-10-5D*, we produced *Arabidopsis* plants overexpressing *TaAP2-10-5D* under the control of the MAS promoter (**Figure 6A**). The expression levels of *TaAP2-10-5D* were detected in 6 T_3_ homozygous transgenic *Arabidopsis* lines by qPCR, and three independent lines with the highest expression levels (L1, L2, and L3) were selected for further analyses (**Figure 6B**).

**Figure 7:**
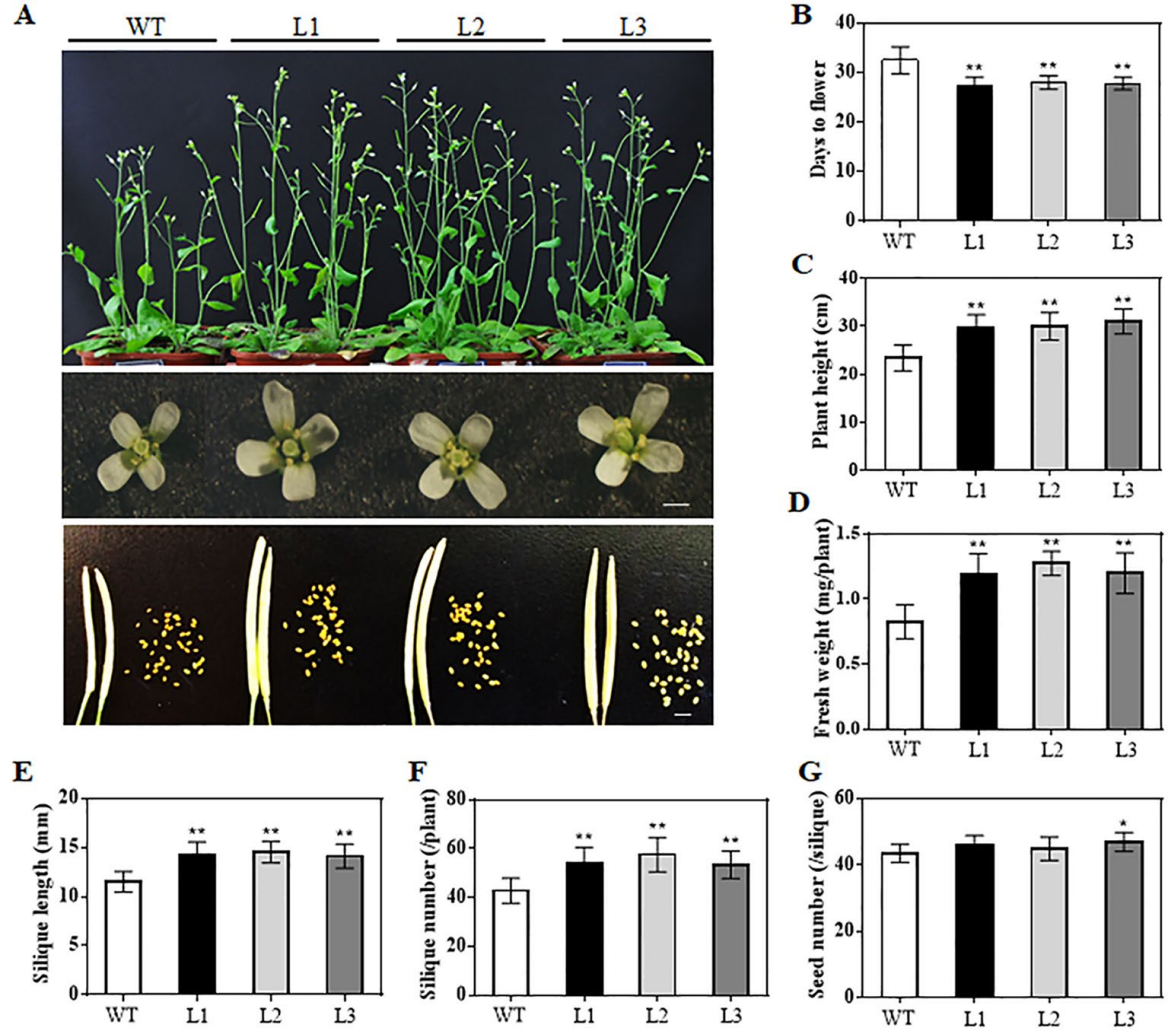
Phenotypic analyses of the *TaAP2-10-5D* transgenic* Arabidopsis* at reproductive stages. **(A)** Comparisons of the whole plants, flower sizes and silique sizes between WT and transgenic plants. Bar = 1 mm. **(B**–**G)** Flowering times **(B)**, plant height **(C)**, fresh weight **(D)**, silique length **(E)**, silique number **(F)** and seed number per silique **(G)** of WT and transgenic plants. Mean values were calculated from 10–20 biological replicates, with error bars representing standard deviations. Statistically significant differences are indicated: *, *P* < 0.05; **, *P* < 0.01 (Student’s *t*-test).

**Figure 8:**
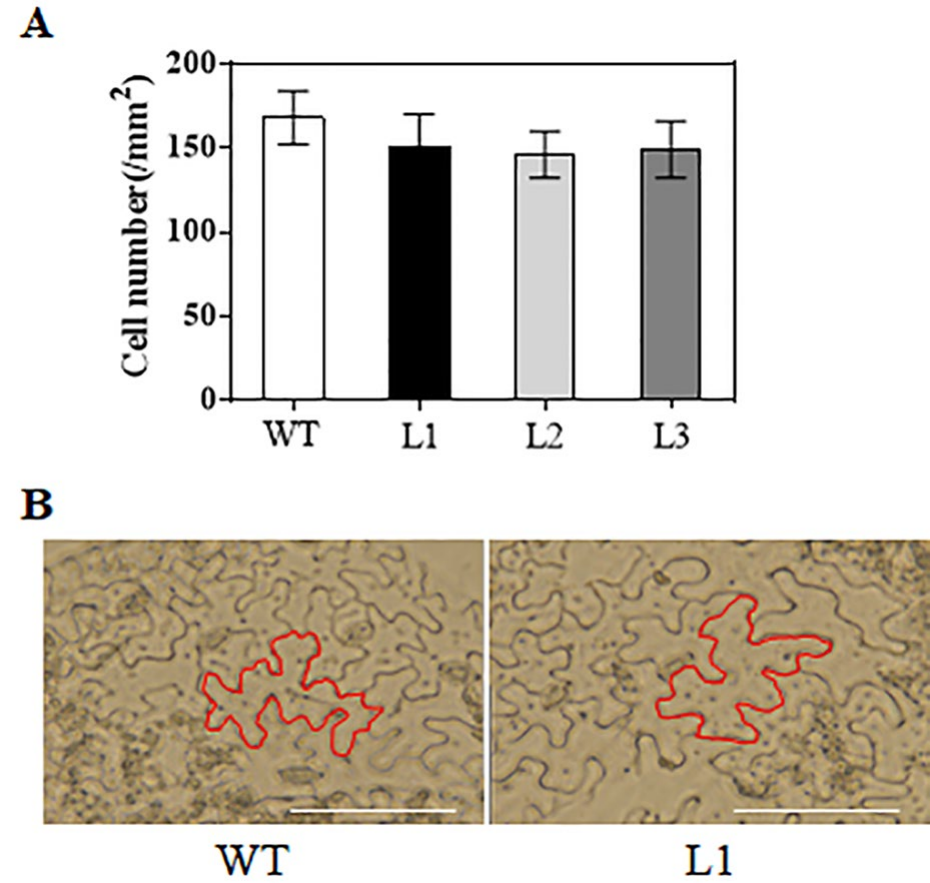
Analysis of cell number **(A)** and cell size **(B)** in *TaAP2-10-5D* transgenic plants. Mean values were calculated from five biological replicates. Bars = 50 μm. Error bars represent standard deviations.

When grown for 7 days, the *TaAP2-10-5D* transgenic seedlings exhibited longer roots than WT plants (**Figures 6C, D**). The seedling size of the transgenic plants at 28 d was significantly larger than that of the WT (**Figure 6C, E**). Additionally, *TaAP2-10-5D* overexpression shortened the flowering time and enlarged the flower dimension in transgenic *Arabidopsis* plants (**Figures 7A, B**). Furthermore, plant height, fresh weight, silique length, and silique number of the transgenic *TaAP2-10-5D* plants were markedly increased compared with those of the WT plants at the mature stage (**Figures 7C–F**). For seed number per silique, no significant differences were observed between transgenic and WT plants (**Figure 7G**). To explore the potential factors leading to the bigger plant size of transgenic plants, we examined the leaf epidermal cell number per unit area and the leaf epidermal cell size on the 5th leaves of the 28-day-old WT and transgenic plants. No striking differences were found for both cell number per unit area and cell size (**Figures 8A, B**), suggesting that the enlarged plant size in the transgenic plants was resulted from cell proliferation.

## Discussion

Genome-wide analysis of a gene family represents an effective approach to the characterization of plant gene functions, and it facilitates the study of the evolution of genes and genomes (Zhang et al., 2015a). In the present study, 62 *AP2* genes were identified from a genome-wide search against the current version of the wheat genome data. Phylogenetic analyses revealed that all the three phylogenetic groups (euANT, basalANT, and euAP2) contained more members in wheat than in *Arabidopsis* or rice. For instance, in the basalANT group, wheat had nearly 10 times as many AP2 genes as their *Arabidopsis* counterparts (**Figure 1** and **Figure S1**), which may be the result of its allohexaploidy and a complex evolutionary process.

Domains and motifs of TFs were proven to be involved in various activities including protein interaction, transcriptional activity, and DNA binding (Liu et al., 1999). Motif analysis revealed 16 conserved motifs in TaAP2 proteins, and motifs 1, 2, 3, 4, 5, and 7 were included in AP2 domains. Among them, motifs 1, 2, 3, and 5 were conserved in all the TaAP2 proteins identified in this study, while motifs 4 and 7 were specific to euANT/basalANT and euAP2 groups, respectively. The motif 4 contains a 10-aa insertion while motif 7 lacks a 10-aa insertion. The10-aa insertion has been reported to be a major difference between euANT/basalANT and euAP2 groups (Kim et al., 2006; Horstman et al., 2014). Another difference is that all the euAP2 proteins contained the unique motif 16, which covered the predicted amino acid sequences of miR172 binding sites (**Figure 2A** and **Figure S4**). The miR172 is known to regulate the euAP2 TFs through transcript cleavage in *Arabidopsis*, rice, and *Brassica napus* (Chen, 2004; Tang et al., 2007; Wang et al., 2019b), suggesting that miR172 binding sites are conserved in euAP2 genes in plants. Further, we demonstrated that motifs 6, 8, and 9 were exclusively contained in the proteins of the euANT group. Noticeably, motifs 4, 8 and 9 cover motifs euANT1 (NSC[K/R][K/R]EGQ[T/S]R), euANT2 (WLGFSLS), and euANT3 (PKLEDFLG), respectively, which were reported to be characteristic of the euANT group (**Figure S2**, Kim et al., 2006). In addition, we found that TaAP2-8, TaAP2-9, and TaAP2-10, which were putative homologs of AtANT, contained a unique motif (motif 14) only present in their sequences (**Figure 2A** and **Figure S5**). Investigation into this motif might reveal new biological functions and regulation mechanisms of *ANT* genes. It has been suggested that TFs that share unique motifs in a cluster are likely to possess similar functions. For example, in the ERF proteins family, the ERF-associated amphiphilic repression (EAR) motif ((L/F)DLN(L/F)XP) was specifically present in the VIII group genes, and it was essentially required for a repression function of ERF proteins (Nakano et al., 2006). In this study, we found that TaAP2-23/24 also had the EAR motif (**Figure S4**). Liu et al. (2018) demonstrated that the EAR motif of Q protein, which is TaAP2-23, is essential for the transcriptional repression activity of Q protein.

Gene structure analysis could provide important information about the gene function and evolution. We found that most *TaAP2* genes shared a similar exon/intron structure within the same phylogenetic group although some differences were also observed (**Figure 2B**). The genes in the basalANT group have more diversified structures than those in euANT and euAP2 groups due to their various numbers of introns. In detail, the basalANT genes had 3 to 7 introns, while in the euANT and euAP2 groups, the genes had 7 to 8 and 8 to 9 introns, respectively. It has been revealed that intron gain or loss is the result of selection pressures during evolution in plants, and genes tend to evolve into diverse exon–intron structures and perform distinct functions (Mattick, 1994; Wang et al., 2016b). Our results imply that gene differentiation might have occurred in the wheat TaAP2 family to accomplish different biological functions under selection pressure during the wheat genome formation and evolution.

Gene duplication is frequently observed in plant genomes, arising from polyploidization or through tandem and segmental duplication (Zhang, 2003). The wheat genome (BBAADD) has undergone two rounds of genome duplication early in its evolution, which led to a complex genome consisting of three related sub-genomes that were derived from three different diploid species (Feldman and Levy, 2012). Among the 24 homologous members of *TaAP2* genes identified in this study, a total of 21, 19, and 22 *TaAP2* genes were found on the A, B, and D sub-genomes, respectively (**Figure 3** and **Table S4**). This result implied that gene loss might have occurred in the wheat AP2 gene family, resulting in the loss of some homologous copies. The retention and distribution of *TaAP2s* on homologous chromosomes provide invaluable information for better understanding the interaction and polyploidization of wheat chromosomes. Furthermore, it is worth noting that there was the least number of *TaAP2* genes on the sub-genome B. This observation strengthens the notion that initial gene loss may have occurred in the B sub-genome following tetraploidy to reduce functional redundancy, and the loss maintained following the formation of the hexaploid wheat ~8000–10,000 years ago (Feldman, 2001; Wang et al., 2016b). One such example is *TaAP2-23*, which is the well-known *Q* gene, the homologous copy of *TaAP2-23* on the B sub-genome was missing (**Table S2**). Previous studies revealed that the *q* allele on wheat homologous chromosome 5B became a pseudogene after allotetraploidization (Zhang et al., 2011). Analysis of gene duplication found that TaAP2-11-4A had high sequence similarities with TaAP2-12-7A and TaAP2-12-7D. This finding was in accordance with previous reports that non-homologous translocation between chromosomes 4A and 7B occurred in hexaploid wheat (Devos et al., 1995; Berkman et al., 2012). Therefore, *TaAP2-11-4A* is probably to be the nonexistent *TaAP2-12-7B*. Except for *TaAP2-11-4A*, no tandem or segmental duplication involving *TaAP2s* was discovered in the wheat genome, suggesting that the expansion of the wheat AP2 gene family was originated from polyploidization. In addition, we found that the *TaAP2* genes on chromosome 4A were reversed with their homologous genes on chromosomes 4B and 4D (**Figure 3**). This result reinforce the previous findings that the modern chromosome 4A arm has been reversed in the hexaploid wheat (Ma et al., 2015).

The expression pattern of a gene is closely correlated with its function. Understanding the tendency of gene expression in different gene clusters will help narrow down candidate genes. To understand the potential functions of AP2 family genes in wheat, we analyzed the expression of these genes in various wheat tissues. As shown in **Figure 4**, the members in the same group had similar expression patterns. For instance, *TaAP2-1*, *TaAP2-2*, and *TaAP2-3*, all belonging to the euANT group, were almost exclusively expressed in the roots, suggesting a potential role in the development of roots. On the other hand, however, there is possibility that these three genes are functionally redundant. A previous report demonstrated that *Arabidopsis* *AIL5*, *AIL6*, and *AIL7* genes were all expressed in developing flowers, and that they had partly overlapping functions with the *AtANT* gene in *Arabidopsis* flower development (Krizek, 2015). In addition, we found that most homologous genes shared a similar expression pattern during development. However, different expression profiles were observed for *TaAP2-7* and *TaAP2-10*, suggesting that these genes might have undergone subfunctionalization or neofunctionalization in the wheat evolutionary process.

Accumulating evidence showed that AP2 family genes play pivotal roles in regulating plant growth and development (Scheres and Krizek, 2018; Dipp-Alvarez and Cruz-Ramirez, 2019; Jiang et al., 2019; Wang et al., 2019a). *Arabidopsis AtANT* was one of the first genes in the family that were functionally characterized; it regulates organ size, floral meristem patterning, and plant defense pathways (Elliott et al., 1996; Krizek et al., 2016). In wheat, three TaAP2 family members, TaAP2-8, TaAP2-9, and TaAP2-10, were grouped into the same cluster with AtANT (**Figure 1**). Among them, *TaAP2-10-5D* had a similar expression pattern with *AtANT* (**Figures 4** and **5D**), thus, *TaAP2-10-5D* was selected for further functional characterization. Overexpression of *TaAP2-10-5D* in *Arabidopsis* enlarged organ sizes *via* increased cell numbers rather than cell sizes (**Figure 8**), implying that *TaAP2-10-5D* positively regulates cell proliferation. This result was consistent with the function of the *AtANT* gene in *Arabidopsis*. It will be interesting to reveal a more detailed function of *TaAP2-10-5D* in wheat as well as the underlying molecular basis, *via* wheat transgenesis and CRISPR/Cas genome editing in combination with modern phenotypic analyses and next-generation sequencing (NGS).

## Data Availability Statement

All datasets generated for this study are included in the manuscript/**Supplementary Files**. 

## Author Contributions

YZ carried out the experiments, analyzed the data, and drafted the manuscript. HB, ZX, and HP provided the idea and instructed the research work. RM and DX provided assistance to perform experiments and collect data. HB revised the manuscript. All authors have read and approved the final manuscript.

## Funding

This work was supported by the National Natural Science Foundation of China (31801363).

## Conflict of Interest

The authors declare that the research was conducted in the absence of any commercial or financial relationships that could be construed as a potential conflict of interest.

## Footnotes

1
http://www.wheatgenome.org/

2
http://plants.ensembl.org/index.html

3
https://www.ncbi.nlm.nih.gov/Structure/cdd/wrpsb.cgi

4
http://smart.embl-heidelberg.de/

5
http://pfam.xfam.org/

6
https://blast.ncbi.nlm.nih.gov/Blast.cgi

7
http://web.expasy.org/compute_pi /

8
https://urgi.versailles.inra.fr/blast_iwgsc/blast.php

9
http://www.plantbreeding.wur.nl/UK/software_mapinspect.html

10
http://www.arabidopsis.org/

11
http://rice.plantbiology.msu.edu/

12
http://gsds.cbi.pku.edu.cn/

13
http://meme-suite.org/tools/meme

14
https://wheat.pw.usda.gov/WheatExp/

15
http://imagej.nih.gov/ij/
