# Supplementary material for: Genome-Wide Identification and Analysis of the AP2 Transcription Factor Gene Family in Wheat (Triticum aestivum L.)
Source: Front Plant Sci. 2019 Oct 11;10:1286. doi: 10.3389/fpls.2019.01286 (PMC6797823; doi:10.3389/fpls.2019.01286)
Supplement: Supplementary file 1 [file DataSheet_1.pdf]

Supplementary Material

Supplementary Figures and Tables

Figure S1. Phylogenetic analyses of AP2 proteins from wheat, *Arabidopsis*, and rice using Maximum-Likelihood (ML) algorithm. The numbers at nodes are bootstrap values after 1000 repetitions.

Figure S2. Sequence alignment of the 24 members of wheat AP2 proteins. One protein sequence was randomly selected from each wheat AP2 family member, and used for the sequence alignment. Red boxes indicate motifs euANT1, 2, 3, 4 and *miR172*-binding motif. The blue lines denote the two AP2 domains.

Figure S3. Sequence logos of the 1-16 motifs generated from the MEME analysis. The overall height of each stack represents the conservation of the sequence at that position. The height of letters indicates the enrichment of that base at each position. The Arabic numerals under the colored letters indicate the position of each residue and the width of the motif. Each color of the letters represents a different type of amino acid residue.

Figure S4. Amino acid sequence alignment of the TaAP2-21/22/23/24 genes. Red boxes indicate EAR motif and motifs 1, 2, 3, 5, 7 and 16. The blue lines denote the two AP2 domains.

Figure S5. Amino acid sequence alignment of the TaAP2-8/9/10 and the *Arabidopsis* ANT gene. Red box indicates Motif 14, and the blue lines denote the two AP2 domains.

Table S1. Primer sequences used in this study.

Table S2. List of *TaAP2* genes identified in wheat.

Table S3. MEME motif sequences in wheat AP2 proteins.

Table S4. Identities between TaAP2 proteins. The identity was estimated when the query coverage was >80%, by nucleotide-nucleotide Basic Local Alignment Search Tool in the National Center for Biotechnology Information databases.

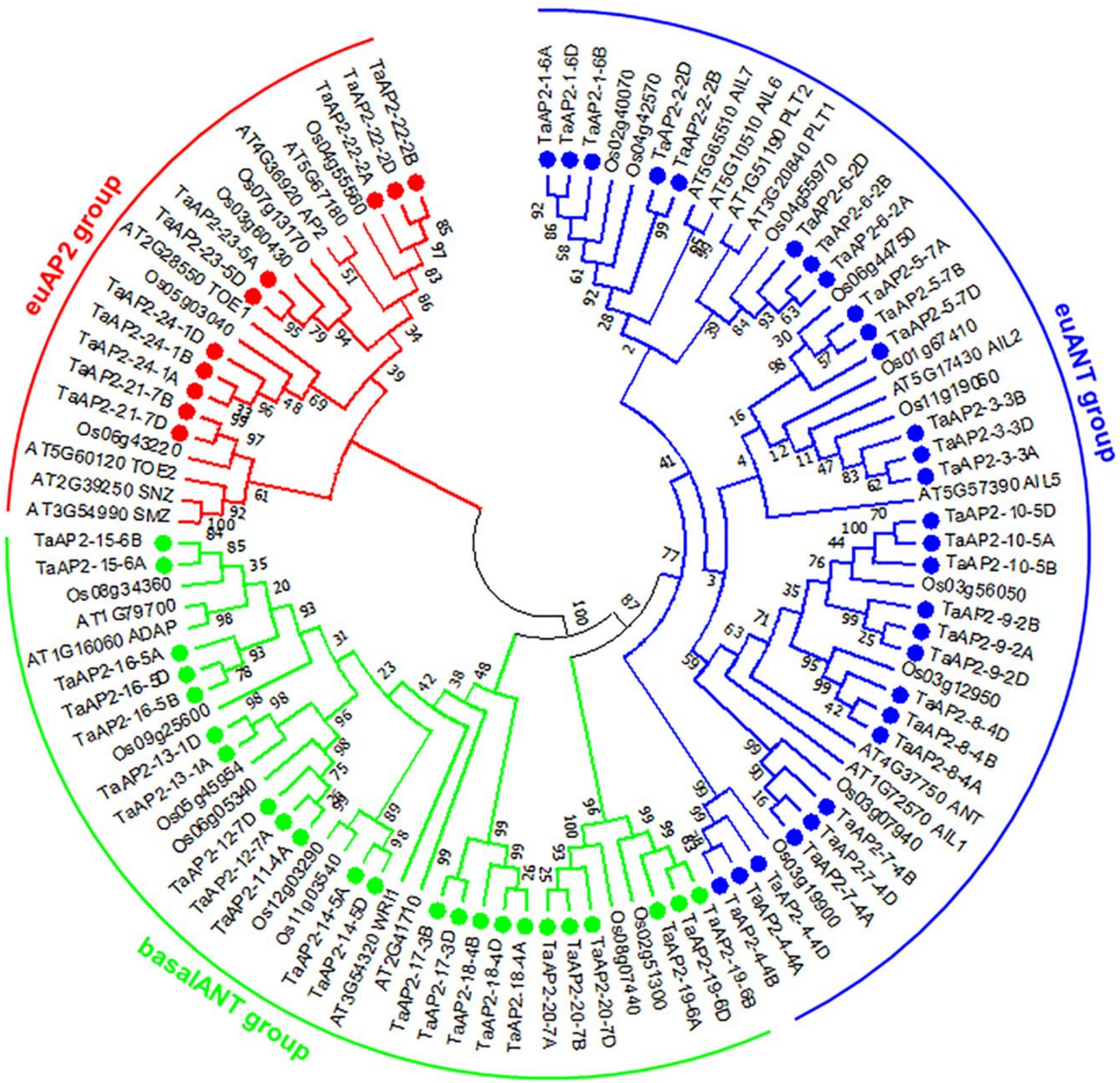

Figure S1. Phylogenetic analyses of AP2 proteins from wheat, *Arabidopsis*, and rice using Maximum-Likelihood (ML) algorithm. The numbers at nodes are bootstrap values after 1000 repetitions.

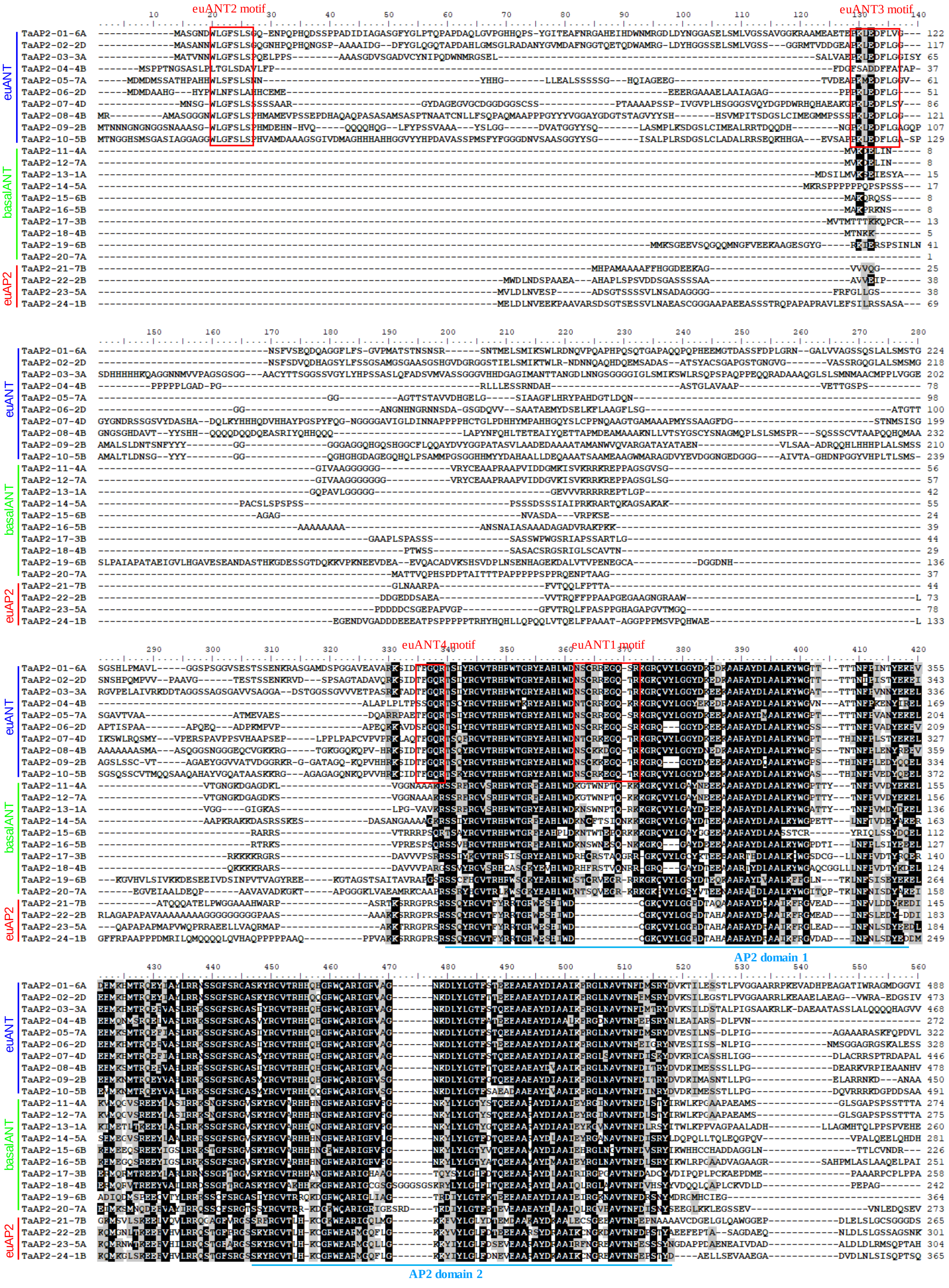
euANT2 motif
euANT3 motif
euANT4 motif
euANT1 motif
AP2 domain 1
AP2 domain 2
euANT
basalANT
euAP2

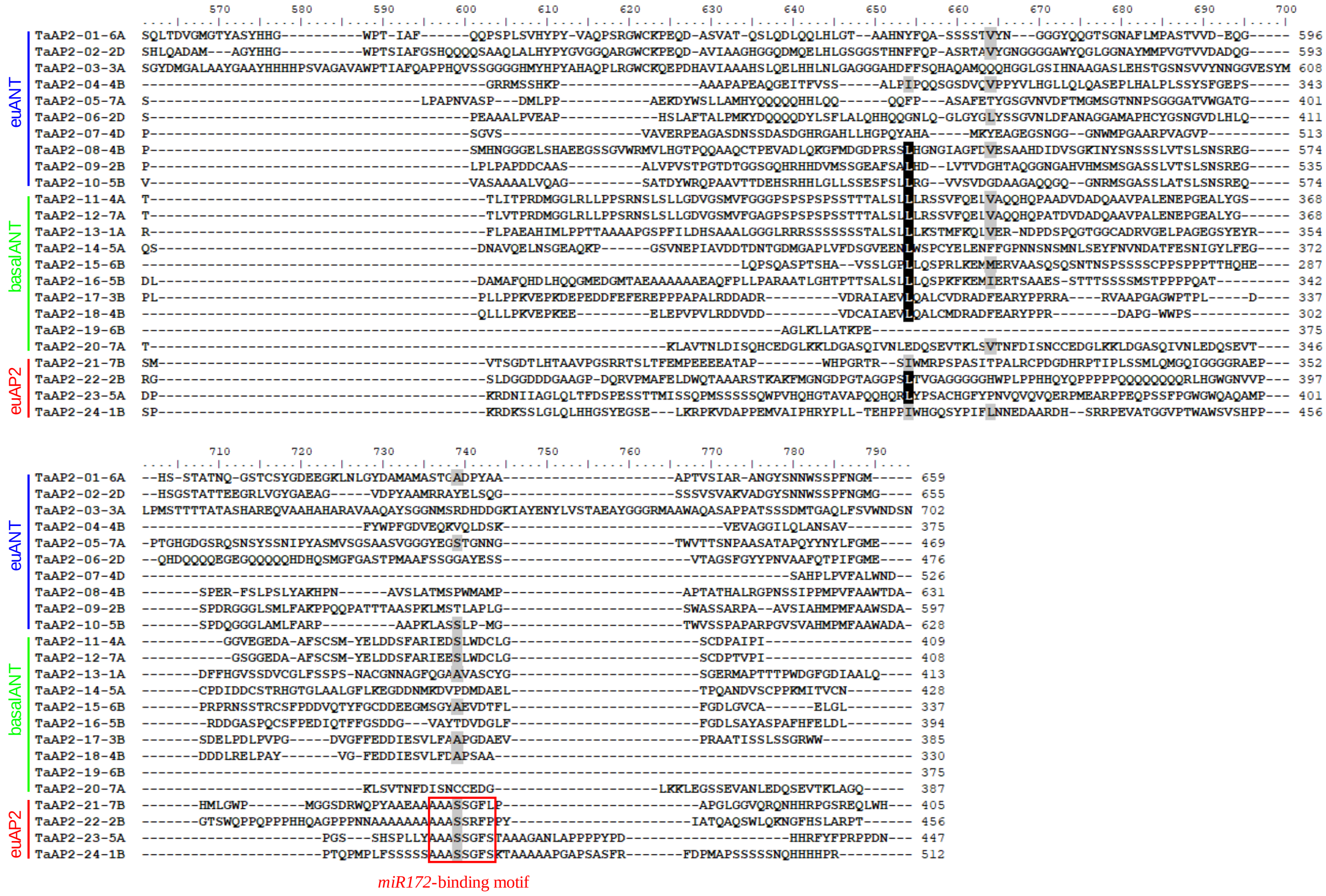

Figure S2. Sequence alignment of the 24 members of wheat AP2 proteins. One protein sequence was randomly selected from each wheat AP2 family member, and used for the sequence alignment. Red boxes indicate motifs euANT1, 2, 3, 4 and *miR172*-binding motif. The blue lines denote the two AP2 domains.

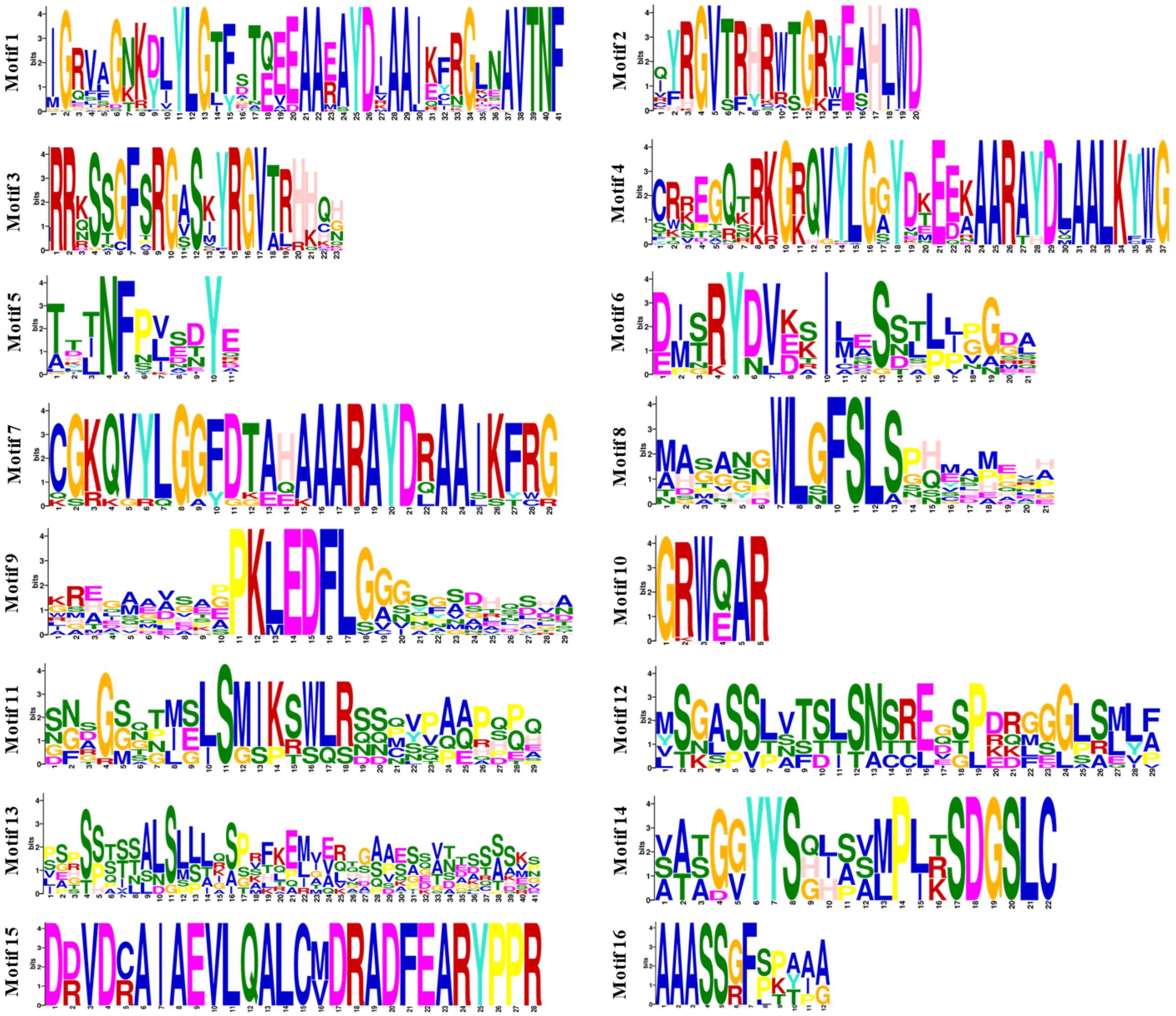

Figure S3. Sequence logos of the 1-16 motifs generated from the MEME analysis. The overall height of each stack represents the conservation of the sequence at that position. The height of letters indicates the enrichment of that base at each position. The Arabic numerals under the colored letters indicate the position of each residue and the width of the motif. Each color of the letters represents a different type of amino acid residue.

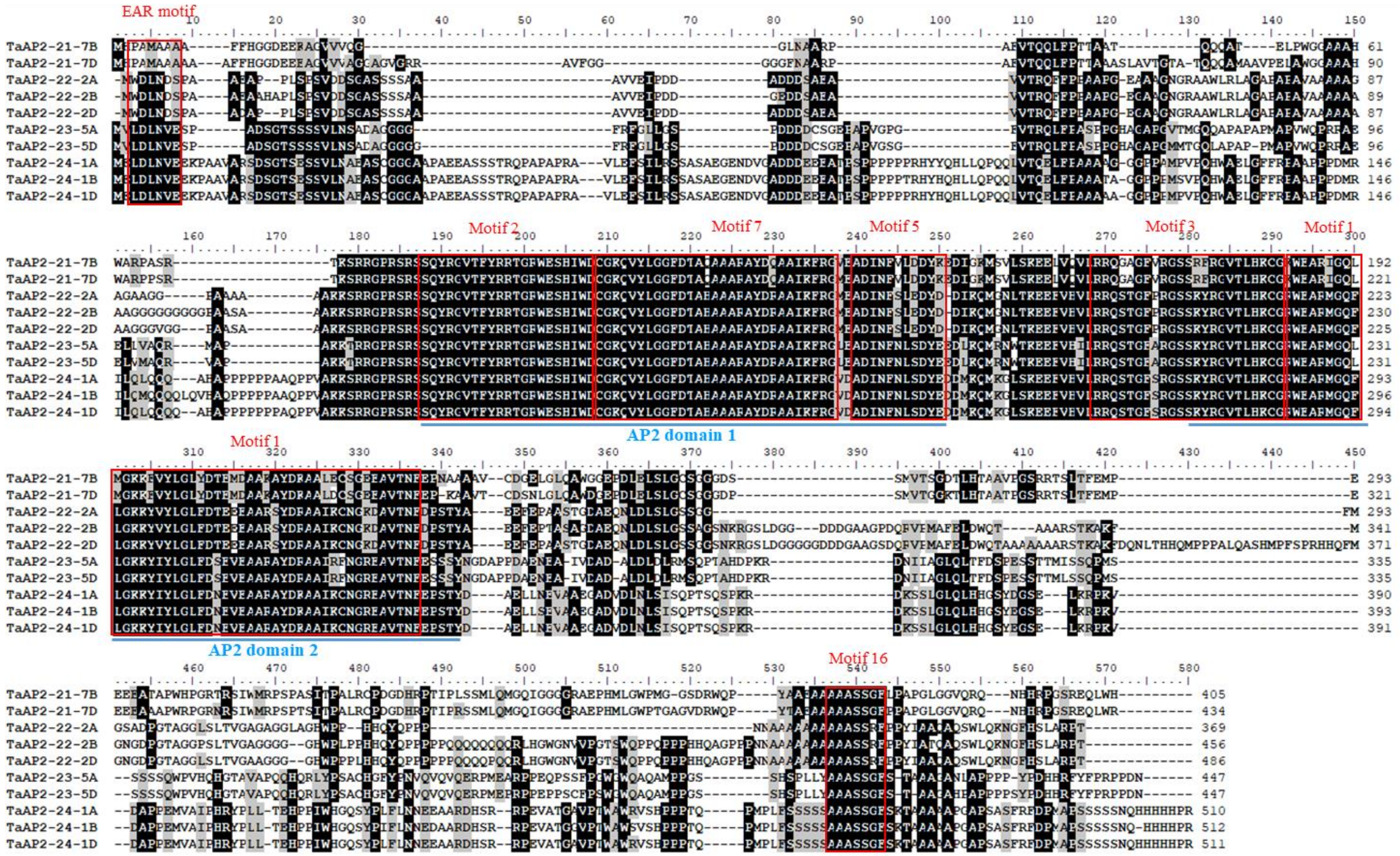

Figure S4. Amino acid sequence alignment of the TaAP2-21/22/23/24 genes. Red boxes indicate EAR motif and motifs 1, 2, 3, 5, 7 and 16. The blue lines denote the two AP2 domains.

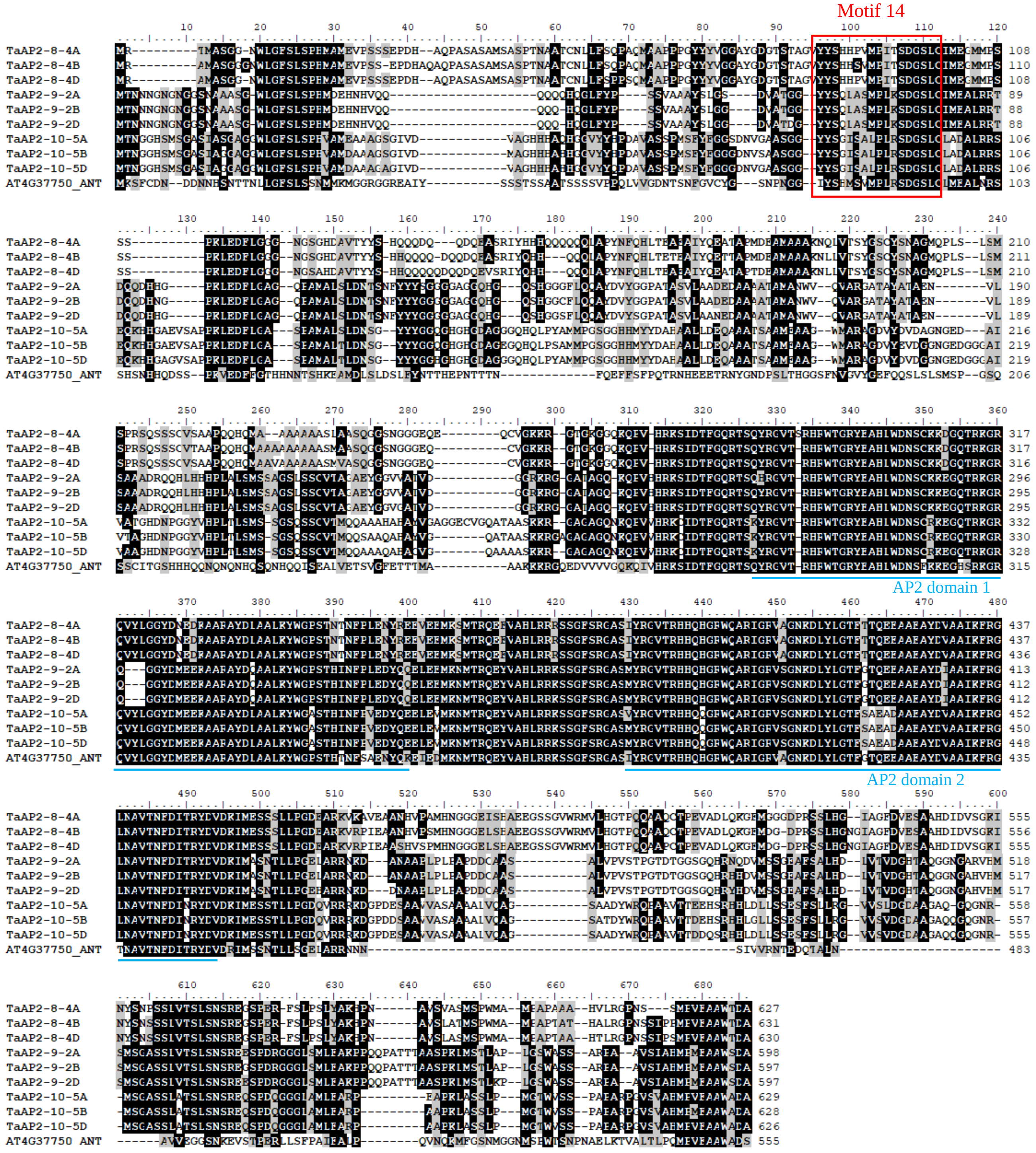

Figure S5. Amino acid sequence alignment of the TaAP2-8/9/10 and the *Arabidopsis* ANT gene. Red box indicates Motif 14, and the blue lines denote the two AP2 domains.

Table S1. Primer sequences used in this study.

Reactions	Primer name	Sequence information	Enzyme site
Gene cloning	TaAP2-10-cF	GGCATCATTGCGACATGTAC	
	TaAP2-10-cR	ACCTTGCTATCTCCTCGTGG	
qPCR	TaAP2-5-qAF	GCCTCCAAGTTCCAACCG	
	TaAP2-5-qAR	CGTCTCAAATGCCGATGCA	
	TaAP2-5-qBF	CTCGGCATTTGACGCCTAT	
	TaAP2-5-qBR	ATCCGGACAGCATGGAGGT	
	TaAP2-5-qDF	CCTCCAAGTTCCAGCCAGAC	
	TaAP2-5-qDR	GGAGCCGTAGGTGTCGAAC	
	TaAP2-8-qF	AGGTGGACTGGGAGATATGAAG	
	TaAP2-8-qR	CAGATCATAAGCCCTGGCAG	
	TaAP2-10-qF	ATGGGCACCTGGGTCTCAT	
	TaAP2-10-qR	ACCTTGCTATCTCCTCGTGG	
	TaAP2-19-qF	GGCACGCATAGGACTGATTG	
	TaAP2-19-qR	CCCTGTCCATGTAGTTGCTTC	
	TaAP2-20-qF	CGCATCCCAGATAGTGAACC	
	TaAP2-20-qR	TTCTGATTGGTCCTCCAGGT	
	TaAP2-22-qF	TCCGCACCACCAATACCAG	
	TaAP2-22-qR	GGAATCGTGATGATGCTGC	
	TaAP2-23-qF	CCTGAATCGTCAACCACAATG	
	TaAP2-23-qR	GCACCTGCACCTGTACGTTC	
	β-action-F	GGAATCCATGAGACCACCTAC	
	β-action-R	GACCCAGACAACTCGCAAC	
Transgenic *Arabidopsis*	TaAP2-10-tDF	ATACACCAAATCGACTCTAGAATGACCA<br>ACGGCGGCCAC	*Xba* I
	TaAP2-10-tDR	CGATCGGGGAAATTCGAGCTCTCACGCG<br>TCGGCCCACG	*Sac* I
Gene expression	TaAP2-10-5D-qF	CCTGTCGAGCGAGTCCTTCT	
	TaAP2-10-5D-qR	ATGAGACCCAGGTGCCCAT	
	Actin2-F	GCTCCTCTTAACCCAAAGGC	
	Actin2-R	CACACCATCACCAGAATCCAGC	

Table S2. List of *TaAP2* genes identified in wheat.

No.	Gene ID	Gene name	Predicted protein length (aa)	Isoelectric point (*p*I)	MW (kDa)	Location	Number of introns
1	TraesCS6A02G229500.1	TaAP2-1-6A	659	5.84	70.6	6A:432258200-432253249	8
2	TraesCS6B02G252000.1	TaAP2-1-6B	662	5.77	70.4	6B:452171125-452177168	8
3	TraesCS6D02G205300.1	TaAP2-1-6D	661	5.74	70.6	6D:291231569-291236698	8
4	TraesCS2B02G378100.1	TaAP2-2-2B	660	6.09	69.7	2B:541006065-541010573	8
5	TraesCS2D02G357600.1	TaAP2-2-2D	655	5.99	69.4	2D:459772278-459776622	8
6	TraesCS3A02G395500.1	TaAP2-3-3A	702	6.67	73.7	3A:642918395-642923286	8
7	TraesCS3B02G427300.1	TaAP2-3-3B	702	6.51	73.6	3B:666443379-666448137	8
8	TraesCS3D02G389100.1	TaAP2-3-3D	703	6.58	73.8	3D:504729631-504734106	8
9	TraesCS4A02G123800.1	TaAP2-4-4A	378	6.8	41.2	4A:156140931-156137580	7
10	TraesCS4B02G180600.1	TaAP2-4-4B	376	7.8	41	4B:395605116-395610375	7
11	TraesCS4D02G182100.1	TaAP2-4-4D	376	6.76	41.1	4D:318288353-318292040	7
12	TraesCS7A02G495800.1	TaAP2-5-7A	469	6.05	50.7	7A:686022222-686019248	8
13	TraesCS7B02G401400.1	TaAP2-5-7B	467	6.01	50.4	7B:667746804-667749818	8
14	TraesCS7D02G483200.1	TaAP2-5-7D	469	6.01	50.6	7D:593239584-593236336	8
15	TraesCS2A02G505600.1	TaAP2-6-2A	478	5.81	51.8	2A:733858560-733853915	7
16	TraesCS2B02G534000.1	TaAP2-6-2B	483	5.95	52.1	2B:730302997-730298363	7
17	TraesCS2D02G506500.1	TaAP2-6-2D	476	5.81	51.4	2D:600318433:600322117	7
18	TraesCS4A02G011600.1	TaAP2-7-4A	571	7.19	61.1	4A:6637867-6634927	7
19	TraesCS4B02G292900.1	TaAP2-7-4B	528	7.75	56.7	4B:578223909:578227846	7
20	TraesCS4D02G291300.1	TaAP2-7-4D	528	7.43	56.4	4D:462747679-462743942	7
21	TraesCS4A02G060400.1	TaAP2-8-4A	626	6.6	67.4	4A:57163080-57167213	7
22	TraesCS4B02G235900.1	TaAP2-8-4B	631	6.6	68	4B:493527066-493531170	7
23	TraesCS4D02G237300.1	TaAP2-8-4D	630	6.6	67.9	4D:399530884-399535156	7
24	TraesCS2A02G267600.1	TaAP2-9-2A	601	6.98	63.9	2A:428908003-428910307	7
25	TraesCS2B02G268100.1	TaAP2-9-2B	600	6.99	63.7	2B:361426266-361422708	7
26	TraesCS2D02G256600.1	TaAP2-9-2D	600	6.86	64	2D:311715470-311719066	7
27	TraesCS5A02G405400.1	TaAP2-10-5A	629	6.5	66.2	5A:597253962-597257923	7
28	TraesCS5B02G410200.1	TaAP2-10-5B	628	6.67	66	5B:585472763-585476653	7
29	TraesCS5D02G415500.1	TaAP2-10-5D	626	6.75	65.6	5D:477983538-477987292	7
30	TraesCS4A02G395400.1	TaAP2-11-4A	409	8.49	43.8	4A:670146027:670148458	4
31	TraesCS7A02G095100.1	TaAP2-12-7A	413	8.49	44.2	7A:57981527-57978393	4
32	TraesCS7D02G091100.1	TaAP2-12-7D	415	7.66	44.4	7D:54439844-54437062	4
33	TraesCS1A02G357800.1	TaAP2-13-1A	413	9.15	44.7	1A:540134248-540135929	3
34	TraesCS1D02G362500.1	TaAP2-13-1D	414	9.15	45	1D:444846239-444845235	3
35	TraesCS5A02G141700.1	TaAP2-14-5A	428	5.64	47	5A:314663869-314668556	6
36	TraesCS5D02G150500.1	TaAP2-14-5D	427	5.46	46.8	5D:239281899-239277231	6

37	TraesCS6A02G125700.1	TaAP2-15-6A	349	9.47	38.4	6A:99062693-99067677	6
38	TraesCS6B02G153800.1	TaAP2-15-6B	337	9.22	37.6	6B:157554399-157559854	6
39	TraesCS5A02G221600.1	TaAP2-16-5A	396	6.43	43.1	5A:437916669-437912671	7
40	TraesCS5B02G220400.1	TaAP2-16-5B	397	6.43	43.2	5B:394747913-394743451	7
41	TraesCS5D02G229400.1	TaAP2-16-5D	396	6.57	43	5D:336426301-336422233	7
42	TraesCS3B02G368400.1	TaAP2-17-3B	385	8.67	42.7	3B:580782561-580785439	6
43	TraesCS3D02G330400.1	TaAP2-17-3D	380	8.71	41.9	3D:442971446-442974166	6
44	TraesCS4A02G181300.1	TaAP2-18-4A	366	6.35	40.3	4A:456290968-456292378	6
45	TraesCS4B02G136400.1	TaAP2-18-4B	333	7	37.1	4B:179850195-179847882	6
46	TraesCS4D02G131200.1	TaAP2-18-4D	328	6.95	36.3	4D:117734913-117732657	6
47	TraesCS6A02G306300.1	TaAP2-19-6A	375	5.28	40.9	6A:540042466-540047792	7
48	TraesCS6B02G334700.1	TaAP2-19-6B	375	5.7	40.9	6B:588831446-588836574	7
49	TraesCS6D02G285400.1	TaAP2-19-6D	381	5.16	41.9	6D:393651353-393656470	7
50	TraesCS7A02G292900.1	TaAP2-20-7A	387	5.25	42.3	7A:370540636-370543677	6
51	TraesCS7B02G186900.1	TaAP2-20-7B	353	5.65	38.4	7B:312443901-312441077	6
52	TraesCS7D02G292500.1	TaAP2-20-7D	351	5.78	38.3	7D:358473231-358476125	6
53	TraesCS7B02G440400.1	TaAP2-21-7B	405	7.71	43.7	7B:705967799-705964977	9
54	TraesCS7D02G512600.1	TaAP2-21-7D	434	9.11	46.4	7D:614168702-614165955	9
55	TraesCS2A02G514200.1	TaAP2-22-2A	473	6.93	50	2A:738439301-738435953	9
56	TraesCS2B02G542400.1	TaAP2-22-2B	484	6.63	51.1	2B:740214920-740218704	9
57	TraesCS2D02G515800.1	TaAP2-22-2D	486	6.6	51.1	2D:606755679-606752894	9
58	TraesCS5A02G473800.1	TaAP2-23-5A	447	6.68	48.9	5A:650130900-650127237	9
59	TraesCS5D02G486600.1	TaAP2-23-5D	450	6.71	49.2	5D:521716648-521712806	9
60	TraesCS1A02G058400.1	TaAP2-24-1A	510	6.58	55.5	1A:38733500-38728652	8
61	TraesCS1B02G076300.1	TaAP2-24-1B	512	6.47	55.7	1B:59192865-59197481	8
62	TraesCS1D02G059200.1	TaAP2-24-1D	496	6.88	53.9	1D:39292323-39287710	8

Table S3. MEME motif sequences in wheat AP2 proteins.

Motif	Width	Best possible match
1	41	IGRVAGNKDLYLGTFSTZEEAAEAYDIAAIKFRGLNAVTNF
2	20	QYRGVTRHRWTGRYEAHLWD
3	23	RRKSSGFSRGASKYRGVTRHHQH
4	37	CRREGQTRKGRQVYLGGYDKEEKAARAYDLAALKYWG
5	11	TTTNFPVSDYE
6	21	DISRYDVKSILESSTLIPGDA
7	29	CGKQVYLGGFDTAHAAARAYDRAAIKFRG
8	21	MASANGWLGFSLSPHMAMEEA
9	29	KREGAAASAEPKLEDFLGGGSGSDHQSHA
10	6	GRWZAR
11	29	SNSGSNTMSLSMIKSWLRSSPVPAAPQPQ
12	29	MSGASSLVTSLSNSREGSPDRGGGLSLLF
13	41	PSPSSTTSALSLLLQSPRFKEMVERQGAAESSVNTSSASKS
14	22	AASGGYYSHLSAMPLKSDGSLC
15	28	DDVDCAIAEVLQALCMDRADFEARYPPR
16	12	AAASSGFPPAAA

Table S4. Identities between TaAP2 proteins. The identity was estimated when the query coverage was >80%, by nucleotide-nucleotide Basic Local Alignment Search Tool in the National Center for Biotechnology Information databases.

% of coverage / % of identity	TaAP2-1-6A	TaAP2-1-6B	TaAP2-1-6D	TaAP2-2-2B	TaAP2-2-2D	TaAP2-3-3A	TaAP2-3-3B	TaAP2-3-3D	TaAP2-4-4A	TaAP2-4-4B	TaAP2-4-4D	TaAP2-5-7A	TaAP2-5-7B	TaAP2-5-7D	TaAP2-6-2A	TaAP2-6-2B	TaAP2-6-2D	TaAP2-7-4A	TaAP2-7-4B	TaAP2-7-4D	TaAP2-8-4A	TaAP2-8-4B	TaAP2-8-4D	TaAP2-9-2A	TaAP2-9-2B	TaAP2-9-2D	TaAP2-10-5A	TaAP2-10-5B	TaAP2-10-5D	TaAP2-11-4A	TaAP2-12-7A	TaAP2-12-7D	TaAP2-13-1A	TaAP2-13-1D	TaAP2-14-5A	TaAP2-14-5D	TaAP2-15-6A	TaAP2-15-6B	TaAP2-16-5A	TaAP2-16-5B	TaAP2-16-5D	TaAP2-17-3B	TaAP2-17-3D	TaAP2-18-4A	TaAP2-18-4B	TaAP2-18-4D	TaAP2-19-6A	TaAP2-19-6B	TaAP2-19-6D	TaAP2-20-7A	TaAP2-20-7B	TaAP2-20-7D	TaAP2-21-7B	TaAP2-21-7D	TaAP2-22-2A	TaAP2-22-2B	TaAP2-22-2D	TaAP2-23-5A	TaAP2-23-5D	TaAP2-24-1A	TaAP2-24-1B	TaAP2-24-1D
TaAP2-1-6A	100	100	100																																																											
TaAP2-1-6B	95	100	100																																																											
TaAP2-1-6D	96	95.4	100																																																											
TaAP2-2-2B				100	100																																																									
TaAP2-2-2D				97.1	100																																																									
TaAP2-3-3A						100																																																								
TaAP2-3-3B						94.1	100	100																																																						
TaAP2-3-3D						94.8	96.6	100																																																						
TaAP2-4-4A									100	99	99																																																			
TaAP2-4-4B									95.9	100	100																																																			
TaAP2-4-4D									95.9	96.2	100																																																			
TaAP2-5-7A												100	100	100																																																
TaAP2-5-7B												94.1	100	100																																															
TaAP2-5-7D												94.4	95.5	100																																																
TaAP2-6-2A															100	100	100																																													
TaAP2-6-2B															95.6	100	100																																													
TaAP2-6-2D															95	95.6	100																																													
TaAP2-7-4A																		100	100	99																																										
TaAP2-7-4B																		95.2	100	99																																										
TaAP2-7-4D																		96.2	97.6	100																																										
TaAP2-8-4A																					100	100	100																																							
TaAP2-8-4B																					93.9	100	100																																							
TaAP2-8-4D																					94	96.4	100																																							
TaAP2-9-2A																								100	100	100																																				
TaAP2-9-2B																								97.4	100	100																																				
TaAP2-9-2D																								98	97.8	100																																				
TaAP2-10-5A																											100	100	100																																			
TaAP2-10-5B																											95.6	100	100																																			
TaAP2-10-5D																											95.5	97.2	100																																			
TaAP2-11-4A																														100	100	100																																		
TaAP2-12-7A																														95.3	100	100																																		
TaAP2-12-7D																														96.3	95.3	100																																		
TaAP2-13-1A																																	100	100																																
TaAP2-13-1D																																	97.6	100																																
TaAP2-14-5A																																			100	100																														
TaAP2-14-5D																																			95.4	100																														
TaAP2-15-6A																																					100	100																												
TaAP2-15-6B																																					87.4	100																												
TaAP2-16-5A																																							100	100	100																									
TaAP2-16-5B																																							95.3	100	100																									
TaAP2-16-5D																																							96.9	96	100																									
TaAP2-17-3B																																										100	100																							
TaAP2-17-3D																																										92	100																							
TaAP2-18-4A																																												100	91	100																				
TaAP2-18-4B																																												96.7	100	100																				
TaAP2-18-4D																																												95.3	96.5	100																				
TaAP2-19-6A																																															100	100	100																	
TaAP2-19-6B																																															96.8	100	100																	
TaAP2-19-6D																																															97.3	96.6	100																	
TaAP2-20-7A																																																		100	100	100														
TaAP2-20-7B																																																		97.3	100	100														
TaAP2-20-7D																																																		98.5	98.6	100														
TaAP2-21-7B																																																					100	92												
TaAP2-21-7D																																																					95.1	100												
TaAP2-22-2A																																																							100	99	92								
TaAP2-22-2B																																																							94.2	100	99								
TaAP2-22-2D																																																							96.7	94.3	100								
TaAP2-23-5A																																																										100	99				
TaAP2-23-5D																																																										96.1	100				
TaAP2-24-1A																																																												100	99	100
TaAP2-24-1B																																																												97.3	100	99
TaAP2-24-1D																																																												98.4	97.9	100
